# Quantifying Aggregated Uncertainty in *Plasmodium falciparum* Malaria Prevalence and Populations at Risk *via* Efficient Space-Time Geostatistical Joint Simulation

**DOI:** 10.1371/journal.pcbi.1000724

**Published:** 2010-04-01

**Authors:** Peter W. Gething, Anand P. Patil, Simon I. Hay

**Affiliations:** Spatial Ecology and Epidemiology Group, Department of Zoology, University of Oxford, Oxford, United Kingdom; University of Michigan and Howard Hughes Med. Inst., United States of America

## Abstract

Risk maps estimating the spatial distribution of infectious diseases are required to guide public health policy from local to global scales. The advent of model-based geostatistics (MBG) has allowed these maps to be generated in a formal statistical framework, providing robust metrics of map uncertainty that enhances their utility for decision-makers. In many settings, decision-makers require spatially aggregated measures over large regions such as the mean prevalence within a country or administrative region, or national populations living under different levels of risk. Existing MBG mapping approaches provide suitable metrics of local uncertainty—the fidelity of predictions at each mapped pixel—but have not been adapted for measuring uncertainty over large areas, due largely to a series of fundamental computational constraints. Here the authors present a new efficient approximating algorithm that can generate for the first time the necessary joint simulation of prevalence values across the very large prediction spaces needed for global scale mapping. This new approach is implemented in conjunction with an established model for *P. falciparum* allowing robust estimates of mean prevalence at any specified level of spatial aggregation. The model is used to provide estimates of national populations at risk under three policy-relevant prevalence thresholds, along with accompanying model-based measures of uncertainty. By overcoming previously unchallenged computational barriers, this study illustrates how MBG approaches, already at the forefront of infectious disease mapping, can be extended to provide large-scale aggregate measures appropriate for decision-makers.

## Author Summary

Reliable disease maps can support rational decision making. These maps are often made by interpolation: taking disease data from field studies and predicting values for the gaps between the data to make a complete map. Such maps always contain uncertainty, however, and measuring this uncertainty is vital so that the reliability of risk maps can be determined. A modern approach called model-based geostatistics (MBG) has led to increasingly sophisticated ways of mapping disease and measuring spatial uncertainty. Many health management decisions are made for administrative areas (e.g., districts, provinces, countries) and disease maps can support these decisions by averaging their values over the regions of interest. Carrying out this aggregation in conjunction with MBG techniques has not previously been possible for very large maps, however, due largely to the computational constraints involved. This study has addressed this problem by developing a new algorithm and allows aggregation of a global MBG disease map over very large areas. It is used to estimate *Plasmodium falciparum* malaria prevalence and corresponding populations at risk worldwide, aggregated across regions of different sizes. These estimates are a cornerstone for disease burden estimation and are provided in full to facilitate that process.

## Introduction

Risk maps estimating the spatial distribution of infectious diseases in relation to underlying populations are required to support public health decision-making at local to global scales [1]–[3]. The advancement of theory, increasing availability of computation and growing recognition of the importance of robust handling of uncertainty have all contributed to the emergence in recent years of a new paradigm in the mapping of disease: the use of a special family of generalised linear models known as model-based geostatistics (MBG), generally implemented in a Bayesian framework [4],[5].

MBG models take point observations of disease prevalence from dispersed survey locations and generate continuous maps by interpolating prevalence at unsampled locations across raster grid surfaces. The most striking advantage of MBG in disease mapping is its handling of uncertainty. Interpolating sparse, often imperfectly sampled, survey data to predict disease prevalence across wide regions results in inherently uncertain risk maps, with the level of uncertainty varying spatially as a function of the density, quality, and sample size of available survey data, and moderated by the underlying spatial variability of the disease in question. MBG approaches allow these sources of uncertainty to be propagated to the final mapped output, predicting a probability distribution (known formally as a posterior predictive distribution) for the prevalence at each location of interest. Where predictions are made with small uncertainty, these distributions will be tightly concentrated around a central value; where uncertainty is large they will be more dispersed. These techniques have been used to generate robust and informative risk maps for malaria [6]–[12], as well as a range of other infectious diseases [13]–[19], at scales varying from national to global. Some studies have extended the handling of variation through space to also include the temporal dimension, allowing disease risk to be modelled and quantified over time as well as space [6],[20].

Implementation of MBG models over even relatively small areas is extremely computationally expensive. Not only are the matrix algebra operations required to generate predictions at each individual pixel costly compared to simpler interpolation methods [21],[22], but this cost must be multiplied many times because prediction uncertainty is evaluated by generating many, equally probable, “realisations” of prevalence at each pixel. Implementations of MBG disease models over large areas therefore tend to be *via* “per-pixel” computation whereby complete maps are built up by generating predictive realisations for each pixel independently. This allows the computational task to be broken down into many small, more easily manageable, operations. Such an approach yields appropriate measures of “local” uncertainty: the set of realisations for each pixel represents a posterior predictive distribution of prevalence from which summary statistics such as the mean, inter-quartile range or 95% credible intervals can be readily extracted, providing the user with valid uncertainty information for each individual location considered in isolation.

There is often a need to evaluate disease prevalence aggregated across spatial regions, temporal periods, or combinations of both [23],[24]. This may be to quantify and compare mean prevalence between countries or administrative units, for example, or to measure a shift in mean prevalence between the start and end of an intervention period or policy change. In addition, MBG prevalence models can be used to estimate derived quantities such as population totals living in regions at different levels of risk, or the burden of disease cases expected within individual countries or continents as a function of underlying prevalence [25], quantities that by definition exist only over aggregated space-time units. It is not possible, however, to construct posterior distributions for these aggregate quantities using a per-pixel approach. To estimate the mean of a region made up of multiple pixels, and the uncertainty around this estimate, the correlation between all the pixels in the region must be known. In a per-pixel approach, each pixel is modelled as independent of its neighbours, ignoring any spatial or temporal correlation. Failing to account for correlation between pixels leads to gross underestimates of the uncertainty in the aggregated quantity, especially over large regions [26].

The solution to the problem outlined above is to replace per-pixel simulation of prevalence realisations with the simultaneous or ‘joint’ simulation of all pixels to be aggregated, recreating appropriate spatial and temporal correlation between them [26]. Crucially, the set of pixel values can then be aggregated in any way, or used as input in derived aggregated quantities, and realisations of these aggregations will have the appropriate posterior predictive distributions. Whilst conceptually simple, the extension from local to regional simulation induces a fundamental computational constraint in that the necessary calculations can no longer be disaggregated into separate tasks for each pixel. This constraint has thus far prevented any use of MBG in disease mapping for the evaluation of aggregate quantities over very large areas, despite the profound public health importance of such measures. Where examples of joint simulation in MBG disease mapping exist, they tend either to be over very small spatial regions [8] or are achieved by simply breaking larger regions down manually into smaller more manageable tiles [17].

In this paper we use a new approximate algorithm for joint simulation to quantify, for the first time, aggregated uncertainty over space and time in a global scale MBG disease model for *Plasmodium falciparum* malaria prevalence [6]. We exemplify how this approach allows uncertainty in prevalence predictions to be enumerated at the continental, national, and sub-national scales at which public-health decisions are usually made. We then extend the model architecture to estimate a second quantity of particular epidemiological interest: national populations at risk (PAR) under different policy-relevant strata of *P. falciparum* transmission intensity.

PAR estimates form a fundamental metric for malaria decision-makers at national and international levels [24],[27] and have also been used to assess equity in donor funding distributions [28], chart the changing exposure of human populations to the disease [29] and provide baselines for predicted changes in exposure under climate change scenarios [30]. A range of techniques have been used to estimate PAR, including the use of MBG and other prevalence models to delineate risk strata in relation to underlying population distributions [6], [29], [31]–[33]. None of these studies have incorporated the inherent uncertainty in prevalence estimates, however, and the resulting PAR estimates are presented as point values with no uncertainty metrics. Here we use the joint simulation framework to generate posterior predictive distributions of PAR living under conditions of low, medium, and high stable transmission within each malaria endemic country, allowing the uncertainty inherent in these estimates to be quantified in a formal statistical framework. These PAR estimates are presented in full with this paper, making them available to any interested parties to support theoretical and applied epidemiological and public health applications.

In the remainder of this introductory section we outline the computational challenges of large scale joint simulation and review existing approaches to overcoming them. In the methods section we present our algorithm for efficient joint simulation over very large grids, detail its implementation and testing with the global *P. falciparum* model, and its extension to estimating PAR. The results section provides the outcome of the testing and validation procedures and examples of jointly simulated realisations of continental, national, and locally aggregated estimates of *P. falciparum* prevalence in 2007. We present our national level estimates of PAR and exemplify how the accompanying uncertainty metrics can be communicated effectively to enhance their utility to decision-makers. We conclude by discussing the strengths and weaknesses of our modelling architecture, the implications for the future of disease mapping, and useful directions for further research.

### The Computational Challenge of Joint Simulation

A general form for MBG models can be defined as follows:
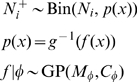
(1)such that in a disease survey of 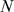 individuals at a given location, the number observed to be infected 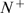 is modelled as binomially distributed with probability of infections given by 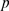, the underlying prevalence of the disease in question, which is modelled as a transformation *via* an inverse link-function 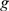 of an unknown Gaussian process (GP) 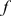 [34],[35]. A Gaussian process in the context of disease mapping is a convenient probability distribution for 2-d surfaces (or 3-d cubes if considering time), describing probabilities associated with different forms of the surface (or cube). Using Bayesian inference, the Gaussian process can be updated to take account of the input data, providing a refined description of these probabilities. Possible surfaces can then be drawn from this updated Gaussian process which, after passing through the inverse link-function, provide realisations of the target disease surface. The Gaussian process can take a wide range of forms: the central tendency at any location is governed by the underlying mean function 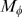, whilst textural properties (the roughness of the surface, and its tendency to fluctuate across space) are governed by the covariance function 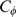. The symbol 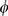 denotes a set of 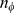 parameters that define the form of the covariance and mean, which can include covariate coefficients.

In MBG, the aim is to estimate the joint posterior distribution of the model parameters 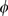 and the values of 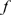 evaluated at all locations and times for which a prediction is required - generally across the nodes of a regular raster grid. Computationally, this task can be split into two distinct phases. Firstly, Markov chain Monte Carlo (MCMC) can be used to generate realisations from the joint posterior of 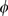 and 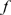 at only the 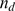 space-time locations 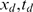 where data exist, denoted 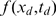. This is intuitive because it is only at these locations that the fit of the Gaussian process is evaluated, and this means the MCMC must only consider a multivariate normal distribution of dimension 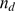, which is generally several orders of magnitude smaller than if all prediction locations across the raster grid were considered. A realisation of 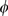 and 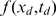 provides a ‘skeleton’ from which the Gaussian process can be evaluated at all prediction locations across a raster grid in a second computational stage. Conditional on these ‘skeleton’ realisations, the value of 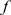 at each prediction location and time 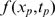 can be sampled from its posterior predictive distribution:
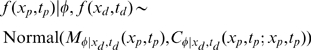
(2)where the posterior predictive mean and covariance parameters are given by the standard conditioning formulas for multivariate normal variables [[36] (p.367)]:
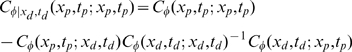
(3)

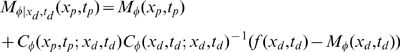
(4)By carrying out this two-step procedure over many realisations, samples are built up from the target posterior predictive distribution 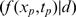.

In a per-pixel implementation, the predictive distributions 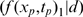, 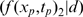, 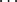 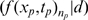 at all 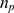 prediction locations in the output raster are realised independently to generate local models of uncertainty. In this case, the largest single computational component is the population and factorisation (*via* a procedure known as the Cholesky decomposition [37]) of the data-to-data covariance matrix 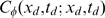 which, in typical disease prevalence data sets where 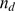 is in the hundreds or thousands, is a relatively minor task that could generally be achieved on a standard desktop computer. The subsequent sampling from the posterior predictive distribution (as in Eq. 2) is trivial: the posterior predictive mean and covariance refer to a single prediction location and sampling therefore amounts to drawing from a univariate normal distribution. Total computation for each pixel is therefore modest, and the cost of generating the maps grows simply in proportion to the number of pixels involved, 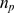.

Switching from a per-pixel implementation to a joint simulation over many prediction locations increases profoundly the computational challenge. The efficiency of a per-pixel approach arises from the effective reduction of 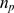 to one, as each pixel is considered in isolation. Joint simulation requires that 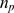 is preserved as the total number of prediction points, which can be many millions if large areas are considered at reasonably fine spatial resolution. In addition to the 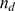 × 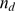 data-to-data covariance matrix, the 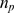 × 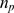 prediction-to-prediction and 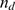 × 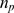 data-to-prediction covariance matrices must be populated. More importantly, in the subsequent sampling from the posterior predictive multivariate normal distribution, the prediction-to-prediction covariance matrix must be factorised [37]. The computational cost of this operation is proportional to the cube of 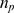. To put this non-linear scaling in context, if a direct joint simulation of a 100×100 raster grid could be computed in one minute, a 1000×1000 grid would take approximately 6×10^7^ seconds (around 694 days). In practice these scaling factors along with those of memory and storage requirements mean direct joint simulation using the equations outlined above is generally limited to predictions at a maximum of around 10,000 points [17],[38], at least two orders of magnitude too few for global scale mapping at sub-10 km resolution, even at a single time period.

### Existing Solutions

In response to the strict computational limits of direct joint simulation outlined above, a wide range of algorithmic and mathematical tools have been developed that increase substantially the maximum number of prediction locations 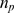 that can be feasibly handled.

The most widely used family of joint simulation algorithms in geostatistics is known as sequential simulation [39]–[41]. Instead of simulating the joint distribution across all 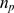 prediction locations simultaneously, sequential simulation evaluates each prediction location in turn. The properties of the multivariate normal model are preserved by conditioning each prediction location not only on the input data, but also on the values simulated at previously evaluated prediction locations, which are effectively treated as conditioning data in subsequent simulations. This approach means the data-to-data covariance matrix 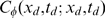 gains an additional row and column after each simulation, ultimately approaching 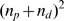 elements, which becomes prohibitively large as 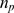 approaches around 10,000 points. In response, sequential simulation algorithms generally limit the conditioning data to a small neighbourhood of 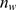 points around each prediction location, specified either by number or by spatial proximity. This computational shortcut is justified by the declining influence of more distant data, which means a simulation conditioned on 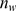 data approximates asymptotically one conditioned on 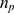 data [39]. Whilst allowing potentially very large prediction grids to be evaluated, the restriction of conditioning data to local neighbourhoods necessitates that, for each prediction location, these 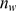 data are identified *via* a search algorithm [40],[42], and bespoke linear algebra systems are evaluated and solved (Eqs. 3 and 4). The cost of the latter for each pixel is proportional to 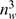, meaning that as the number of prediction locations 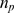 grows, the size of 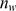 that can be feasibly computed reduces sharply.

In a disease mapping context, the goal is to generate joint simulations conditioned by observed prevalence or incidence data. This precludes the direct use of a wider class of algorithms developed for unconditional joint simulation [5], where the goal is simply to realise random fields with correct mean and covariance properties, unconstrained by any observations. Conditional simulation can, however, be split into an unconditional joint simulation and a per-pixel prediction task [[42] (p.494),[43]], as follows:
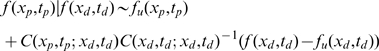
(5)where 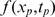 is the target conditioned field. In practical terms, this decomposition allows generation of the conditioned field in two stages: unconditional simulation is used to generate the unconditioned field (the first term above, 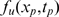), which is then combined with the ‘skeleton’ of the conditioned field at the data points 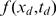 in a standard per-pixel prediction (the second right-hand term). The sum of these terms yields a conditioned field with an identical distribution to one generated directly *via* conditional simulation. The advantage in working with unconditional simulation algorithms arises because, in the absence of irregularly located data, all computations relate to locations arranged in a regular grid, a geometric convenience that can be exploited in a variety of ways [44]–[47]. An elegant and widely used family of techniques for grid-based unconditional simulation is based on spectral decompositions, principally the fast Fourier transform, of which the ‘circulant embedding’ algorithm is particularly popular [48]–[50]. These techniques offer extremely efficient exact simulations but become infeasible for more than around one million prediction locations [38], due in part to memory requirements resulting from the necessary replication of large covariance matrices. Furthermore, such algorithms require the prediction grid to be regular, that is, for pixels to be arranged in rows and columns of equal spacing, which is not the case with global grids defined using spherical coordinates.

## Methods

### A Model-Based Geostatistical Model for *P. falciparum* Parasite Rate

A new global map of *P. falciparum* endemicity in 2007 has recently been published [6], the first such enumeration of global malaria risk in 40 years. This map was generated from an assembly of 7,953 community parasite surveys collated from 78 countries between 1985 and 2007 used with a Bayesian space-time MBG model to predict urban-adjusted *P. falciparum* parasite rate in the epidemiologically informative 2 up to 10 yr age range, *Pf*PR_2–10_, across a regular spherical grid within the limits of stable transmission [31]. The model form is described in full elsewhere [6]. The original implementation of this model used an MCMC inference stage to generate 500 samples from the joint posterior distribution of the space-time Gaussian process at the 7,953 locations for which input parasite rate survey data existed 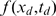, and of a 13 element parameter vector, 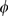. A per-pixel approach was then used to evaluate, for each realisation, values of the Gaussian process at all desired prediction locations 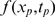, which were then combined with an independently sampled Gaussian random noise component, and subjected to an inverse logit transform and multiplication with an age-correction factor to yield the target quantity *Pf*PR_2–10_. The set of realisations of *Pf*PR_2–10_ for each pixel provided an appropriate measure of local uncertainty, with which the precision of *Pf*PR_2–10_ predictions could be assessed at all individual pixel locations worldwide.

The aim of the current study was to implement the predictions described above *via* joint simulation, allowing quantification of uncertainty in predicted *Pf*PR_2–10_ over spatially and temporally aggregated regions. This presented an unprecedented challenge in geostatistical disease modelling for a number of reasons. Firstly, the target prediction space was exceptionally large: a grid of resolution equivalent to 5×5 km at the equator spanning the extent of stable *P. falciparum* transmission in Africa (the largest contiguous region of interest), evaluated temporally for each of the 276 months of 1985–2007 (the study period of interest, corresponding to the temporal span of the collated *Pf*PR survey assembly) constituted approximately 623 million individual prediction locations, several orders of magnitude larger than any other MBG disease model extent in the published literature. Secondly, the model had a relatively complex form, particularly in the covariance function [51] which was spatiotemporal (covariances were modelled between locations spread across time as well as space), spatially anisotropic (covariance between spatial locations was influenced by direction as well as separation distance), and included a periodic component in the temporal axis (to address observed seasonality). Finally, spatial locations of data and predictions were represented on a sphere, with their separations evaluated using great-circle distance, a geometric complication that was necessary to avoid the distorting effects of map projections when dealing with global scale phenomena. Together, these factors precluded the use of the existing approaches to joint simulation described above. Spectral decomposition-based algorithms for unconditional simulation would have required the data-to-data covariance matrix to be reflected along three axes, exceeding memory limits of currently available computers. More fundamentally, the incorporation of the curvature of the earth in the arrangement of prediction locations meant the matrix could not be considered to be in block Toeplitz form [38],[44],[48]. Whilst a standard sequential simulation could, in principle, have been achieved within available memory constraints, the very large number of prediction locations would have meant limiting conditioning data to insufficiently small prediction neighbourhoods in space and time in order to achieve computation in a feasible timescale. Instead, a novel approximate algorithm for joint simulation was developed that overcame these constraints, and this is presented in the next section.

### Achieving a Very Large Joint Simulation

Sequential simulation algorithms maintain feasible memory and computation requirements over large grids by limiting conditioning data to small local neighbourhoods, but the repeated identification of local data, evaluation of local covariance matrices, and subsequent linear algebra calculations are prohibitively inefficient for very large numbers of prediction locations. The extremely efficient algorithms developed for unconditional joint simulation over regular grids, such as circulant embedding, also reach memory limits for very large prediction tasks, and are not suited to sphere-based grids. In this study a new algorithm was developed that adopted and extended the principle of traditional sequential simulation - that joint simulation over very large areas can be broken down into many small simulations conditioned on nearby values - but incorporated some of the efficiencies exploited by unconditional algorithms operating on a grid whilst overcoming the complications of sphere-based grid systems.

Firstly, the decomposition of a conditional joint simulation into an unconditional joint simulation and a per-pixel conditioning stage was exploited (Eq. 5). The bulk of the computational challenge therefore lay in generating unconditioned realisations of the zero-mean Gaussian process 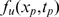 across the 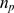 nodes of the 3-d space-time prediction grid given only realisations of the scalar parameter vector 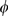, where 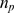 = 6.23×10^8^ was the largest individual prediction task (for the Africa region, with 1718 columns, 1315 rows and 276 months). Each grid pixel was 0.04165 decimal degrees in height and width, corresponding to approximately 5×5 km at the equator. Defining a regular grid in terms of spherical coordinates meant that the width of pixels varied with latitude. A second stage was then required to condition the field 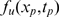 given realisations of the field at the 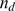 data locations 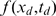 These two stages are now discussed in more detail.

#### Stage 1: Unconditional simulation of a random field

In this first stage the principle of sequential simulation was adapted. Rather than randomly visiting individual prediction locations in turn, each column of each monthly surface of the 3-d space-time prediction grid was jointly simulated in sequence, scanning left-to-right across each monthly surface and from the earliest month (January 1985) to the latest (December 2007). Each simulated column then became available for conditioning subsequent columns. As with conventional sequential simulation, the size of the conditioning set was prevented from becoming prohibitively large by limiting conditioning data to a set of local prediction locations. A key inefficiency in the use of local conditioning neighbourhoods in conventional sequential simulation algorithms is that, because the spatial (or spatiotemporal) configuration of data with respect to the target prediction location in each neighbourhood is likely to be unique, the data-to-data and data-to-prediction covariance evaluations must be repeated for every sequential prediction. In this algorithm, however, the locations of conditioning data relative to the prediction column were defined *a priori*, using a “conditioning footprint”. This footprint prescribed those previously simulated grid locations that were used to condition subsequent columns, identified by their relative rather than absolute position in relation to the prediction column. This allowed a single footprint (once edge-effects had been handled, discussed later) to be used repeatedly to define the location of conditioning pixels for every column.

This procedure resulted in a number of computational advantages over conventional sequential simulation. Firstly, since the locations of conditioning data, 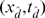, (where the “hat” denotes previously simulated values subsequently treated as conditioning data) were prescribed *a priori*, there was no requirement to repeatedly search for and identify conditioning data proximal to prediction locations. Secondly, because the relative positions of conditioning data were the same for all columns, the associated data-to-prediction 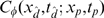, prediction-to-prediction 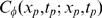, and data-to-data 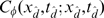, covariance matrices only needed to be evaluated once for each realisation, along with the factorisation of the latter matrix required in the evaluation of the posterior predictive covariance (Eq. 3) and mean (Eq. 4). Defining these components *a priori* meant that computations to be performed per realisation for each column were restricted to a single matrix multiplication to complete the evaluation of the posterior predictive mean (Eq. 4) and the subsequent joint sampling of the column pixel values from the multivariate normal posterior predictive distribution (Eq. 2), resulting in very large savings in memory and computation.

Because the algorithm scanned left-to-right, only columns to the left of the prediction column could be included from the same month. Preceding months could include columns both to the right, directly ‘below’ and to the left. The exact configuration of the footprint in terms of the number and spacing of preceding columns and the number and spacing of preceding months could be varied. Similarly, the density of included columns could be varied such that pixels from every 2nd, 5th, or 10th row of each column, for example, could be included rather than from every row. Analogous to the specification of conditioning neighbourhoods in conventional sequential simulation, the success of the procedure presented here was dependent on a suitable configuration of the conditioning footprint. This configuration represented a trade-off between the computational cost of the algorithm, which scaled sharply with larger and more dense footprints, and the extent to which the resulting unconditioned field approximated the hypothetical result obtained using a direct simulation. The appropriate tool for identifying a suitable footprint configuration was the extent to which the resulting simulated field reproduced the required covariance properties specified by 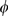, and this is discussed further in subsequent sections.

**Figure 1:**
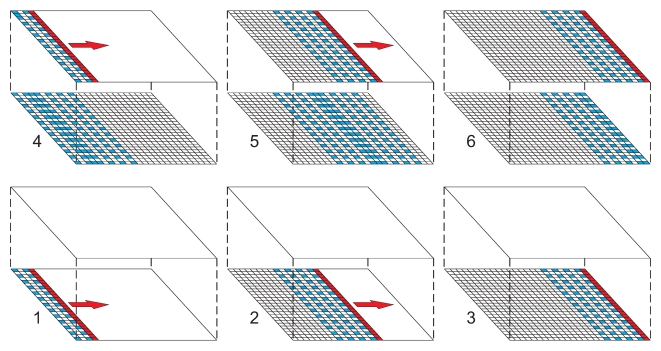
Progression of footprint algorithm for efficient joint simulation of the space-time Gaussian random field. The sequence of schematic diagrams shows the algorithm at six different stages. In this schematic, the prediction space is 25 columns by 25 rows by two months. In each diagram the target column to be predicted is marked in red, pixels already predicted to the left or below the target column are shaded, those yet to be predicted are left white. The ‘footprint’ of conditioning data used in each prediction is shaded blue. In this example the full footprint extent is specified to include, in the target month, seven columns to the left of the target column and, in the preceding month, seven columns to the left, seven to the right and the column directly below the target column. This full extent is thinned to include only every second column and row. Diagram 1 (lower left) shows the algorithm at an early stage: having already simulated values in the first three columns of the first month, the target column being simulated is the fourth from left. The full footprint is truncated and consists of only two columns to the left of the target column. As the algorithm scans across this first month, more columns become available to the left and the footprint grows (diagrams 2 and 3). In diagram 4 the algorithm has moved to the second month, and the footprint can now begin to include simulated pixels from the preceding month. In diagram 5 the full footprint is shown, truncated neither to the left nor right. As the algorithm scans further to the right to complete the second month, the footprint becomes truncated once more, this time by the right-hand margin (diagram 6).

A final algorithmic complication arose from the constraints placed on the footprint configuration by the spatial and temporal boundaries of the grid. Clearly a footprint using 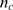 columns to the left of the prediction column is truncated by the left-hand boundary of the grid until the algorithm scans to predict the 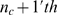 column. A similar truncation occurs as the algorithm approaches the right-hand grid boundary and applies also to the temporal dimension as columns from the preceding 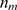 months are not available until the prediction month progresses to month 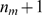. These edge effects were handled by simply defining a series of truncated footprints that gradually became larger as the algorithm scanned away from the left-hand margin, and from the early months. Similarly, as the scan-line approached the far right-hand margin and/or the later months, it again became progressively constrained. Prediction of the very first column, which by definition had no previously simulated data on which to be conditioned, was achieved simply by direct unconditional simulation. The geometric operation of the algorithm is illustrated schematically in Figure 1. Each unique footprint resulted in a corresponding unique data-to-data and data-to-prediction covariance matrix. As with the matrices supporting the non-truncated footprint, these were evaluated and factorised *a priori*, and were stored in memory to be available as the algorithm scanned across the columns of the grid. The up-front memory requirements of the algorithm were therefore heavily influenced by the number of different truncated footprints that needed to be defined, which in turn was determined by the spatial and temporal extent of the full footprint.

#### Stage 2: Generation of a conditioned field

Values of the unconditioned field 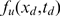 at each of the 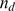 data locations were defined by assigning the values of the nearest prediction node in space and time (leading to a maximum spatial mismatch of <2.5km which was considered acceptable in the global-scale model). A standard per-pixel prediction was then carried out to combine these values with the conditioning data 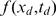 and the unconditioned field 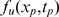, to generate realisations of the target conditioned field 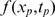 as outlined in Eq. 5.

All code was written in the R [52] and Python [53] programming languages, incorporating only open-access libraries, and is available to download freely from http://github.com/malaria-atlas-project/mbg-world/tree/generic.

### Predicting Aggregated Uncertainty in *P. falciparum* Endemicity Predictions

Having implemented the algorithm described above, the jointly-simulated conditioned field 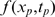 was combined with per-pixel samples from an uncorrelated Gaussian noise component, subject to an inverse-logit transform, and multiplied by an age correction factor to yield realisations of *Pf*PR_2–10_. Crucially, in contrast to the original per-pixel implementation [6], each realisation represented a joint simulation of prevalence, so the back-transformed and age-corrected space-time cube could be aggregated into any arbitrary spatial, temporal, or space-time unit, with realisations of the aggregated quantity representing samples from the posterior predictive distribution. This was exemplified by generating realisations of mean *Pf*PR_2–10_ across the 12 months of 2007 for three scales of spatial aggregation: continental, national, and at the first sub-national administrative unit level, quantities that span the spectrum of information scales required by malaria public-health decision-makers.

### Predicting Aggregated Uncertainty in Populations at Risk Under Different Strata of *P. falciparum* Endemicity

Previous approaches to estimating PAR have used modelled surfaces of *P. falciparum* prevalence to delineate the boundaries of various risk strata, and combine these mapped boundaries with population maps to calculate the population living in each strata [6]. Because the prevalence modelling in this earlier work was set in a per-pixel framework, spatial uncertainty in the prevalence predictions could not be propagated into the PAR estimates since the latter is a spatially aggregated quantity. This limitation was removed in the current study since prevalence was modelled using a joint simulation framework. Population data [54] were obtained and adjusted to form a 1×1 km grid surface for 2007, and a previously defined stratification [31] was used to delineate areas in which stable transmission of *P. falciparum* malaria was likely to occur (defined as areas where incidence is likely to exceed 0.1 case per 1000 per annum). These inputs are explained further in Protocol S1. Within these limits of stable transmission, each jointly simulated realization of *Pf*PR_2–10_ was converted into a categorical map identifying pixels where prevalence was predicted as either low stable (*Pf*PR_2–10_≤5%), medium stable (*Pf*PR_2–10_>5%≤40%) or high stable (*Pf*PR_2–10_>40%) transmission. These prevalence classes have been proposed previously as of particular relevance to decision-makers when developing optimal strategies for intervention and control [55],[56]. Each realized endemicity class map was downscaled to a 1×1 km grid and combined with the population grid and an additional grid identifying national boundaries to allow calculation of a realization of PAR in each of the three endemicity classes in each country. Repeating this procedure across all 500 realizations allowed posterior predictive distributions to be constructed, from which the posterior mean was extracted as a point estimate for each country-class and the inter-quartile range was extracted as an accompanying uncertainty metric.

### Testing and Validation

#### Generating aggregated sets for validation

In the original implementation of the global *P. falciparum* map [6], two aspects of the model performance were tested: the ability (i) to accurately predict *Pf*PR_2–10_ at individual pixel locations and, (ii) crucially, to provide posterior predictive distributions of *Pf*PR_2–10_ that represented appropriate measures of local uncertainty for each pixel. Correspondingly, the joint simulation implemented in the current study was tested for the ability to (i) accurately predict mean *Pf*PR_2–10_ over aggregated sets of pixels and (ii) generate appropriate posterior predictive distributions of these aggregated means. A hold-out set of 10% of the data was selected using a spatially-declustered stratified random sampling procedure described previously [6], and the modelling procedure was re-run using the remaining 90% of data to predict, *via* joint simulation, values at the hold-out locations. Aggregated sets of different sizes were made from this set of jointly simulated predictions. The ideal aggregated sets for testing would have consisted of complete spatial and temporal regions. Such test sets cannot exist, however, since it is both practically and theoretically impossible to measure *Pf*PR_2–10_ continuously over large spatial regions and time periods. As an alternative, sets were made by aggregating non-contiguous pixels from the hold-out set dispersed through space and time. These sets were made by simple random sampling from the full hold-out set and consisted of 1000 sets each of sizes between 2 and 100 pixels. Sets made in this way were dispersed in both space and time and this was preferred to an alternative strategy of defining spatial or temporal-only sets so that the full space-time functionality of the algorithm could be assessed. Additionally, very few long time series of data existed at the same spatial location, preventing the definition of time-only validation sets. For each set, the true arithmetic mean *Pf*PR_2–10_ was extracted, along with the corresponding posterior predictive distribution of the mean generated *via* joint simulation.

#### Testing point predictions of aggregated mean *Pf*PR_2–10_

For each simulated set, a point estimate of the mean *Pf*PR_2–10_ was derived using the mean of the posterior predictive distribution, and the error between this prediction and the observed true mean *Pf*PR_2–10_ was calculated. A plot was constructed that plotted for each set the error value on the *y*-axis and the set size (number of aggregated pixels) on the *x*-axis, allowing a visualisation of the spread of errors associated with sets of different sizes. Additionally, the mean error and mean absolute error were calculated for each set size and overlaid for reference on the same plot.

#### Testing posterior predictive distributions of aggregated mean *Pf*PR_2–10_

The following procedure was implemented to assess the fidelity of the posterior predictive distributions of mean *Pf*PR_2–10_ for each set as models of uncertainty. Firstly, each distribution was summarised using 100 equally spaced quantiles. Secondly, each quantile was considered in turn and the proportion of true mean *Pf*PR_2–10_ values, across all the aggregated sets, that exceeded the corresponding predicted mean *Pf*PR_2–10_ value for that quantile was calculated. This proportion was interpreted as an “observed” probability threshold and was plotted against the “predicted” probability threshold associated with that quantile. In a perfect model it would be expected, for example, that 50% of the true set means would exceed the values predicted by the corresponding 0.5 quantile of each posterior predictive distribution, 90% would exceed the values predicted by the 0.1 quantile, and 99% the value predicted by the 0.01 quantile. By calculating the actual proportions of true means exceeding each of the 100 quantiles, a “coverage” plot was generated that compared these observed and predicted probability thresholds across the range of probabilities from zero to one. In a perfect model, all plotted values would lie on the 1∶1 line indicating an exact correspondence between predicted and observed probability thresholds, and an exact representation of the uncertainty in aggregated predictions. This procedure was carried out for 1000 sets each of size 1, 2, 5, 10, 15, 20, 30, 40, and 50 pixels drawn by simple random sampling from the full space-time hold-out set.

## Results

### Testing and Validation

#### Testing point predictions and posterior predictive distributions of aggregated mean *Pf*PR_2–10_

**Figure 2:**
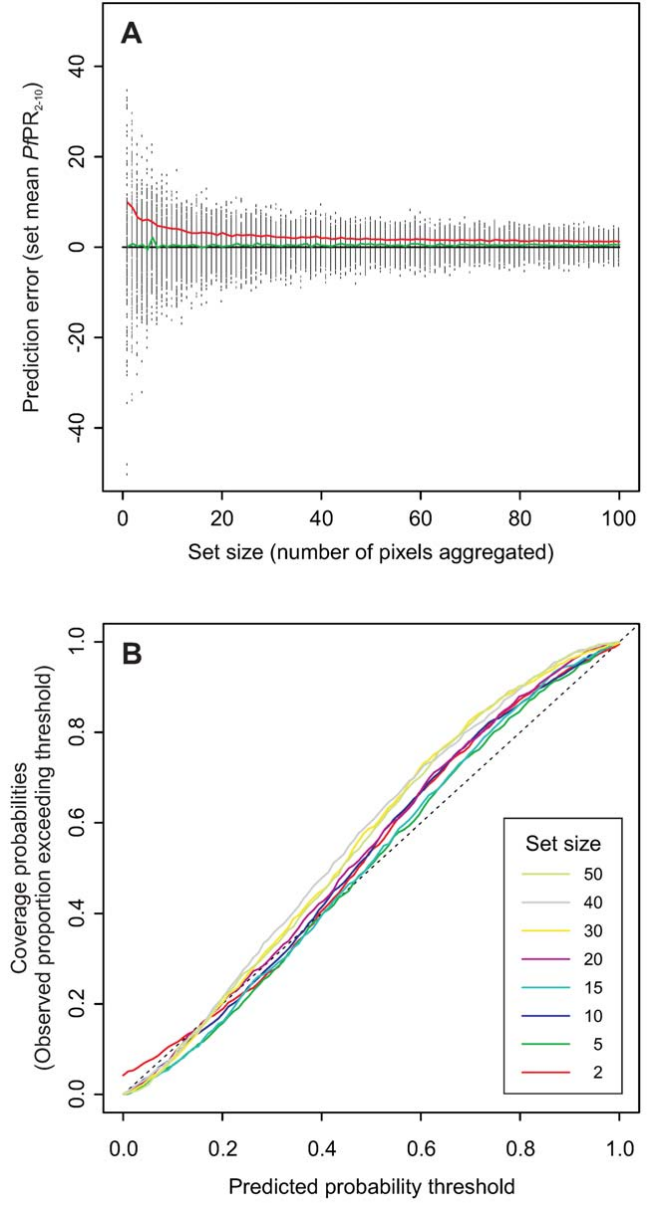
Validation results. A validation procedure generated many sets of aggregated pixels for which a posterior predictive distribution and point estimate of the set-mean *Pf*PR_2–10_ could be compared to the true value. In (A) the error between the point estimate and true value is plotted against the size (number of pixels) of each aggregated set (black dots). Also shown are smoothed moving averages of the mean error (green line) and mean absolute error (red line) in relation to set size. (B) is a coverage plot comparing, for aggregated sets of different sizes, the correspondence between predicted probability thresholds (as provided by the modelled posterior predictive distributions of mean *Pf*PR_2–10_ in the validation sets) and actual probability thresholds (defined as the observed proportions of true set means exceeding the predicted threshold values).

Figure 2(A) shows the errors in the predicted mean *Pf*PR_2–10_ values of many simulated sets of different sizes. The mean error, shown by the green line, was effectively zero for all set sizes, indicating that the model produces unbiased predictions with no overall tendency to over- or under-predict mean *Pf*PR_2–10_. As would be expected, the dispersion of these errors reduced with set size, as did their mean magnitude (shown by the mean absolute error line in red). Mean absolute error for sets of size 1, 25, 50 and 100 pixels was 11.4, 2.7, 1.9 and 1.3 *Pf*PR_2–10_ respectively. Figure 2(B) shows the coverage plots for sets of different sizes. Comparison of predicted and observed probability thresholds revealed a similar pattern for aggregated sets regardless of their size. For probability thresholds between 0 and 0.5, the predicted and observed probabilities corresponded closely. Between thresholds of 0.5 and 1, the predicted probabilities tended to under-predict slightly the observed probability. This meant, for example, that around 80% of the true mean *Pf*PR_2–10_ values exceeded the predicted 0.7 probability threshold, rather than the 70% predicted by the model. This suggested that the rising limb of the posterior predictive distributions tended to rise too gently towards the peak, thus underestimating slightly the probability of a given prediction taking values smaller than its point estimate. Taken as a whole, the coverage plot suggested that posterior predictive distributions were likely to represent predictive uncertainty reasonably well, albeit with slight deviations from a perfect model discussed above.

### Predicting Aggregated Uncertainty in *P. falciparum* Endemicity Predictions

**Figure 3:**
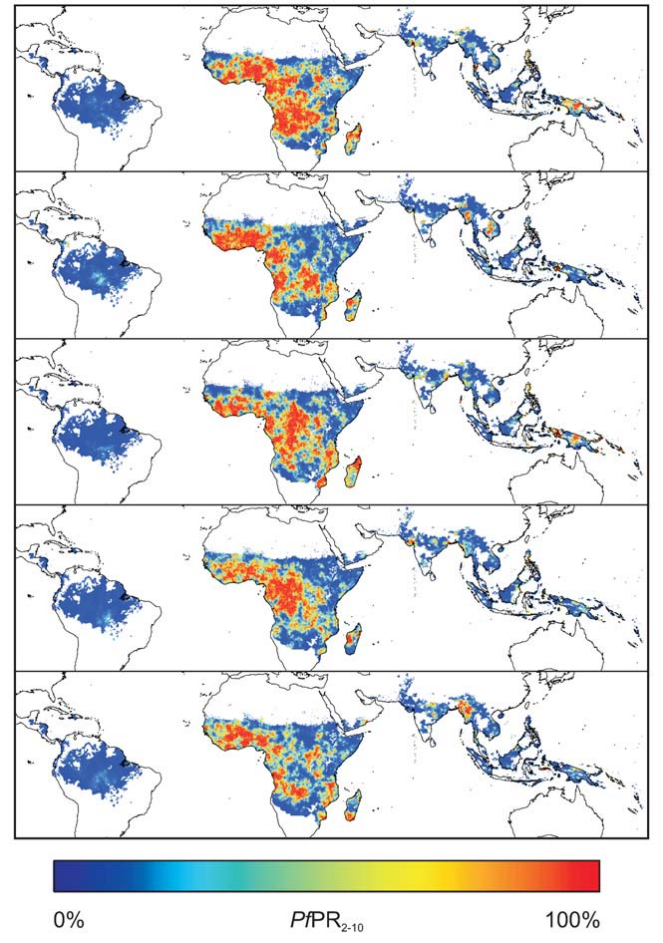
Simulated global surfaces of *Pf*PR_2–10_. Examples of five of the 500 realisations of *Pf*PR_2–10_ generated *via* the joint simulation algorithm. Each of these maps represents an equally possible ‘reality’ and the full set of 500 provides a model of the probable prevalence at all locations. Because each map is jointly simulated, pixels within any spatial region can be aggregated together to define a regional mean, and the 500 different versions of that mean across the set of maps provides a model of the uncertainty for that mean value. Simulation is constrained to the global limits of stable transmission.

**Figure 4:**
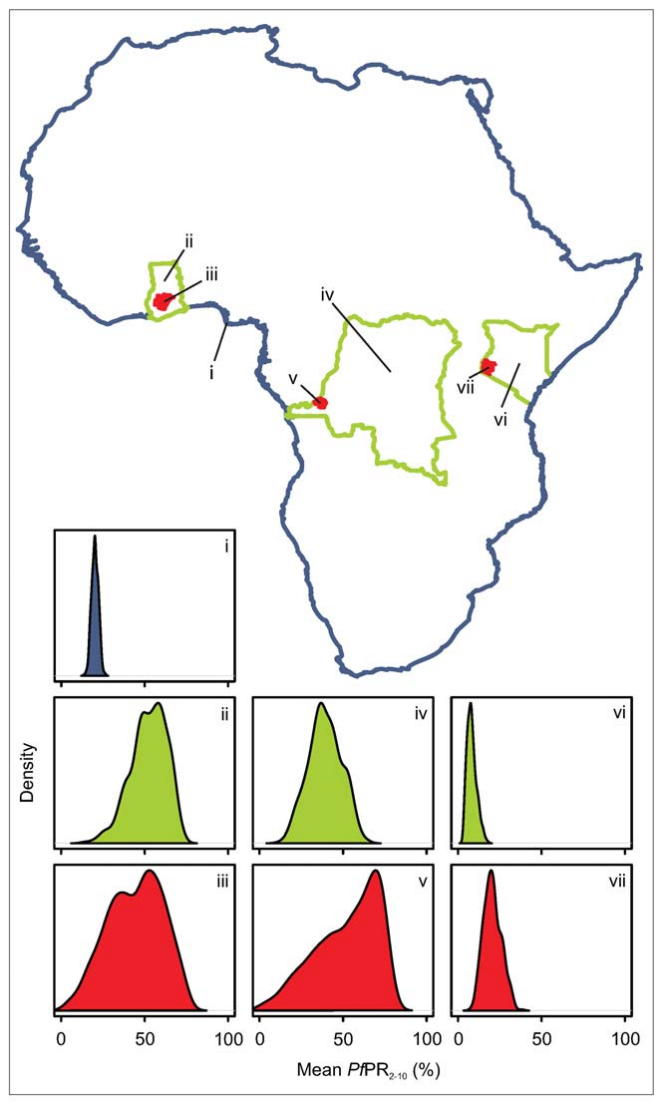
Simulated posterior predictive distributions of *Pf*PR_2–10_ at different spatial scales. The crucial feature of the jointly simulated realisations of *Pf*PR_2–10_ was that they could be aggregated over arbitrary spatial and/or temporal regions to generate posterior predictive distributions of mean *Pf*PR_2–10_, constituting appropriate models of regional uncertainty. In this example, such predicted distributions are provided at three different scales, predicting mean *Pf*PR_2–10_ for the entire African continent (i); across the nations of Ghana (ii), Democratic Republic of Congo (iv) and Kenya (vi); and across a first administrative level unit of those countries (Ashanti Region, Ghana(iii); Kinshasa Province, DRC (v); Nyanza Province, Kenya (vii)).

Figure 3 provides examples of five of the 500 jointly simulated realisations of *Pf*PR_2–10_ within the global limits of stable transmission, aggregated temporally across the 12 months of 2007. None of these maps, taken individually, are intended to represent the true pattern of global prevalence. Each is driven by the underlying data but represents a random draw from a universe of possible maps given the model specification, the information in the data, and the resultant modelled uncertainty. Whilst the large-scale regional patterns of endemicity are similar in each realisation, small scale heterogeneity exists between each, and this variation across the 500 realisations defined the form of the posterior predictive distribution of the global surface. The validation procedures explained above provided evidence of the suitability of these surfaces to be aggregated spatially or temporally to provide appropriate posterior predictive distributions of mean *Pf*PR_2–10_ within spatiotemporal units of different sizes. Figure 4 provides examples of this functionality: posterior predictive distributions are shown for mean *Pf*PR_2–10_ across the entire African continent, across three individual countries (Ghana, Democratic Republic of Congo (DRC) and Kenya), and across a first level administrative unit in each of these countries (Ashanti Region, Ghana; Kinshasa Province, DRC; Nyanza Province, Kenya). As would be expected, the dispersion of the distributions, which can be interpreted directly as the modelled uncertainty in the predicted mean *Pf*PR_2–10_, tended to decrease as aggregated predictions were made over progressively larger regions, such that the continent-wide mean was predicted with lower uncertainty than were national-level means which, in turn, were less uncertain than first-level administrative unit means. Dispersion was also moderated, however, by predictive uncertainty influenced by the availability of input survey data in different regions. This explains why the posterior predictive distribution for DRC, a country with very few available survey data, is substantially more dispersed than that for Kenya, for which many survey points exist, despite constituting a much larger spatial unit of aggregation. These example plots also illustrate in general terms why joint-simulation is necessary when predicting aggregated prevalence. Under a standard per-pixel implementation with all locations simulated independently, the variance of the aggregated mean *Pf*PR_2–10_ would decline in proportion to the square-root of the number of pixels in the aggregated unit. At even the first administrative unit level, this would result in artificially small variances for the posterior predictive distributions. At national and continental levels the predicted uncertainty would effectively be zero. Under the joint simulation approach presented here, the space-time variance structure is preserved and this resulted in even the continent-wide prediction retaining a non-negligible level of uncertainty.

### Predicting Aggregated Uncertainty in Populations at Risk under Different Strata of *P. falciparum* Endemicity

**Figure 5:**
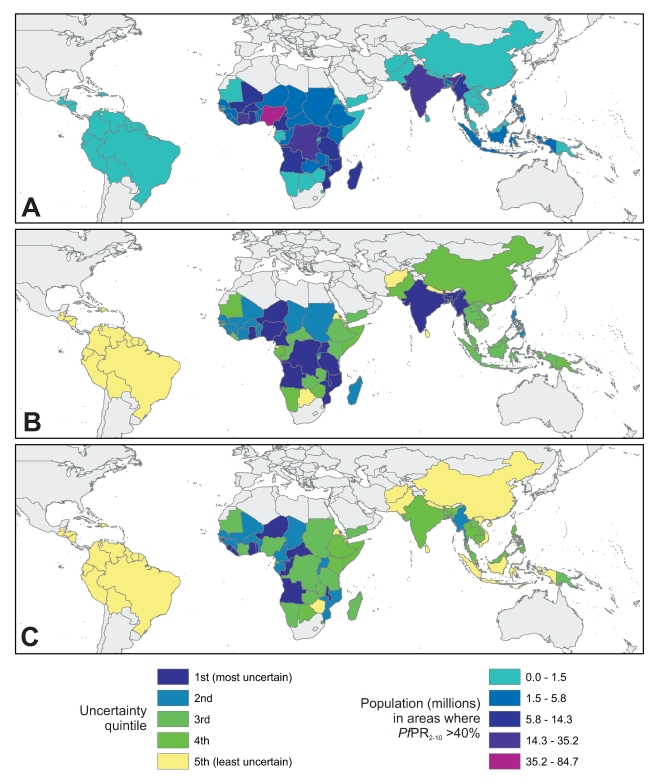
Predicted national populations at risk of high stable transmission and associated ranked uncertainty metrics. Map A shows the estimated population at risk of high stable transmission (*Pf*PR_2–10_>40%) for each of the 80 *P. falciparum* malaria endemic countries considered, based on the mean of each posterior predictive distribution. Map B shows the ranked uncertainty associated with each of these national estimates, quantified using the width of each posterior inter-quartile range, and ordered into quintiles such that countries in quintile one have the largest uncertainty and quintile five the smallest. Map C presents the same uncertainty metric but based on national population proportions in this risk class rather than absolute numbers.

Estimated 2007 populations living under low, medium, and high stable transmission risk are presented by country in Protocol S1 along with accompanying posterior inter-quartile ranges. Figure 5(A) provides an example of mapped national PAR estimates for the high stable transmission risk class (*Pf*PR_2–10_>40%). Figure 5(B) shows a ranking of each country in terms of model-based uncertainty, quantified by comparing the width of the posterior inter quartile range associated with each PAR estimate. Figure 5(C) shows an equivalent uncertainty ranking for relative PAR (percentages of each country's population living under high stable conditions). Further details on the methods and interpretation of the presented results can be found in Protocol S1.

## Discussion

Numerous algorithms exist that seek to increase the efficiency of joint simulation, including the widely-used family of sequential simulation algorithms, and those based on spectral decompositions operating on regular lattices. Whilst these elegant algorithms expand considerably the magnitude of joint simulation tasks that can be achieved relative to a direct calculation, none of them could produce simulations on arbitrary input grids on the scale required for global-scale disease maps as addressed in this study. We have overcome these limitations using a practical elaboration of standard sequential simulation that is empirically highly efficient but does not put any special requirements on the input grid or covariance function. The approach represents an important increase in the feasibility of aggregated uncertainty assessment over very large prediction spaces, expanding the scope of geostatistical models in global scale epidemiology.

The current study builds on a modelling framework for the global mapping of *P. falciparum* prevalence defined in an earlier study [6]. Like many large-scale MBG disease mapping studies published to date, this earlier work presented prevalence maps with per-pixel uncertainty metrics that could not be used to define uncertainty around aggregated prevalence predictions. Similarly, this per-pixel approach did not support the evaluation of uncertainty around important derived aggregate quantities such as populations at risk. By setting this earlier model in a joint simulation framework, the current study allows the formal prediction of aggregated *P. falciparum* prevalence and national populations under different prevalence strata with appropriate measures of uncertainty. Figure 5 shows these national PAR estimates for populations living under conditions of high stable transmission. A prevalence threshold of *Pf*PR_2–10_>40% has been proposed as separating lower transmission settings, where universal coverage of insecticide treated bed-nets could interrupt transmission [56], from higher transmission settings where this coverage alone would be insufficient and scale-up of additional interventions would be required to achieve elimination [55],[56]. Under these recommendations, the quantification of PAR in this high stable transmission class has direct implications for national resource requirements as very large numbers of people in these nations will require more than universal coverage of bed-nets to interrupt transmission.

The direct practical utility to decision-makers of accompanying uncertainty metrics is less well established since they have not been available previously. Uptake by decision-makers will be aided by the packaging of uncertainty measures into easily understood information and the ranked uncertainty maps presented in Figure 5 (B and C) highlight one such approach. Recognising those countries where high-risk populations can be identified with least certainty provides a basis for rational deployment of global surveying, monitoring, and evaluation efforts for populations that will carry the largest burden of global malaria morbidity. Figure 5(B) illustrates that the least certain countries are, as would be expected, those which include areas of high transmission risk in conjunction with very large human populations, such as India, Myanmar, DRC and Nigeria. Figure 5(C) considers population proportions at risk and therefore standardises for absolute population size. This removes, amongst others, India, Nigeria and DRC from the set of least certain countries and adds some smaller high risk nations such as Togo, Liberia and Sierra Leone.

In this study we have presented the extension of a jointly simulated prediction framework for *P. falciparum* prevalence to estimates of PAR. The framework could readily be extended to the prediction of related aggregate quantities of substantial public health significance. An important example is the prediction of *P. falciparum* clinical case incidence which can be estimated empirically as a function of prevalence [25] and the underlying population density [54]. By basing these estimates on the jointly simulated prevalence predictions presented here, incidence estimates can be summed to provide national or continental-level estimates with appropriate credible intervals. A second important measure is the basic reproductive number, *R*_0_, which provides biological insight into the intensity of malaria transmission and is particularly useful when assessing the effect of current or future interventions [57]–[59]. Again, *R*_0_ can be estimated as a function of prevalence [59],[60] and the jointly simulated surfaces presented here can be incorporated with these models to provide country level estimates of *R*_0_ useful for strategic planning.

The approach presented in this study can be applied readily to any large-scale MBG prediction of infectious disease prevalence and corresponding populations at risk. An important caveat for further applications, however, is that the algorithm cannot be treated as a black-box that will generate appropriate output without user supervision. The algorithm relies on a key assumption: that the use of a relatively small proportion of conditioning data proximate to each target prediction column generates predicted values that are sufficiently similar to a theoretical (although infeasible) direct joint simulation based on all locations simultaneously. In reality, the footprint-based predictions will approach the theoretical “true” values asymptotically, such that the use of progressively more conditioning data in the footprint will result in progressively smaller increases in convergence between the two sets of values. This leads to a delicate trade-off between feasible computational demand and appropriate predictive precision. Suitable resolutions of this trade-off cannot be prescribed *a priori* since they rely on factors that will vary between settings. On one hand, the sparsest permissible footprint will be determined by the space-time covariance structure of the disease measure under study. On the other, the computational demand will scale non-linearly with the size of the study area and the spatial and temporal resolution at which it is to be modelled. In this study, appropriate footprint configurations were identified by systematically evaluating the empirical covariance functions of realisations generated under progressively sparser footprint configurations, and the necessary diagnostic scripts are freely available from the authors. In principle, appropriate configurations could be approximated in advance using the target covariance structure parameters, although the implications of the latter on the appropriateness of different configurations is likely to be complex and non-linear. Users of the algorithm should recognise that failure to consider these factors appropriately could lead to misleading or erroneous results.

Automatic optimization of the footprint would be a useful area for future research. Approaches to this problem, based on evaluation of the Markov properties of 2-d fields, have been proposed [61]–[63] although as yet these have not been extended to the 3-d setting necessary for space-time simulation. A number of recent advances in computational infrastructure have emerged that also warrant further investigation in the context of efficient joint simulation over very large grids. In particular the re-purposing of graphic processing units (GPUs) to support extremely efficient parallel processing, and their application to matrix calculations, offers potential decreases in processing time of several orders of magnitude [64] for the current rate-limiting steps of the algorithms presented here: the population and factorisation of large covariance matrices.

The expansion of MBG in epidemiology has been rapid and led to major advances in the handling of uncertainty in disease risk maps. To date, fundamental computational constraints have precluded the use of such models for predictions of aggregated prevalence and populations at risk required by decision-makers across national and continental spatial scales. In this study we have designed, implemented and tested a new algorithm that overcomes the prohibitive computational barriers of large scale joint simulation allowing, for the first time, appropriate handling of aggregated uncertainty in global scale disease maps. This epidemiological insight has been extended to defining national populations at risk with appropriate confidence intervals which are released here in the public domain to support informed efforts in disease burden estimation.

## Supporting Information

Protocol S1 Populations at risk under different levels of *Plasmodium falciparum* malaria intensity.
(0.41 MB DOC)
