# Supplementary material for: Quantifying Aggregated Uncertainty in Plasmodium falciparum Malaria Prevalence and Populations at Risk via Efficient Space-Time Geostatistical Joint Simulation
Source: PLoS Comput Biol. 2010 Apr 1;6(4):e1000724. doi: 10.1371/journal.pcbi.1000724 (PMC2848537; doi:10.1371/journal.pcbi.1000724)
Supplement: Protocol S1 — Populations at risk under different levels of Plasmodium falciparum malaria intensity. (0.41 MB DOC) [file pcbi.1000724.s001.doc]

**Protocol S1: Populations at risk under different levels of *Plasmodium falciparum* malaria intensity**

This supplementary section provides full tabulated output of the populations at risk estimates described in the main manuscript, along with an overview of the methods that generated these tables and further details to aid interpretation.

**S1.1 Methodological overview**

*Defining baseline populations*

The Global Rural Urban Mapping Project (GRUMP) alpha version provides gridded population surfaces for the year 2000, based on worldwide assemblies of national census data augmented with earth observation data such as night time lights images to provide the most reliable and precise contemporary estimate of population counts in every 1 × 1 km pixel across the inhabited land surface [1]. Each pixel value was projected to a 2007 population value by applying national level medium variant inter-censal growth rates by country provided by the United Nations [2], using methods described previously [3].

*Delineating risk-free, unstable and stable transmission areas*

87 countries have been identified previously as being endemic in 2007 for *Plasmodium falciparum* malaria [4] (*P. falciparum* malaria endemic countries, *Pf*MECs). Seven of these countries (South Africa, Saudi Arabia, Belize, Panama, Iran, Kyrgyzstan, Tajikistan) were excluded from the current analysis because they are of universally very low risk of *P. falciparum* malaria and have health information systems capable of enumerating accurately the annual number of cases experienced nationally. As such, the rationale for estimating populations at risk in these countries is strongly diminished and case reports form a more appropriate metric for surveillance. For the remaining 80 countries, populations at risk under varying levels [5] of transmission intensity were estimated in a number of stages, as follows.

Work to stratify all *Pf*MECs into three broad classes of risk has been presented previously [4]. In brief, confirmed *P. falciparum* clinical case data were assembled for 41 of the 80 *Pf*MECs. These data were mapped to first, second, or third administrative level units and used to classify areas as risk free (zero cases), and either unstable or stable risk if the number of confirmed cases was lower or higher than 0.1 per 1,000 people per annum respectively. A simple biological model that identified areas where low temperatures were likely to preclude transmission was used to identify further risk free areas, and a second model incorporated the limiting effect of high aridity on the survival of locally dominant *Anopheles* vectors to downgrade risk in extremely dry regions.

Having stratified each *Pf*MEC into areas of no, unstable, and stable transmission risk, these regions were overlaid with the 2007 GRUMP-derived population surface to obtain estimates of population living under each risk strata for each country, and these values along with the total population form the first four columns for each country row in tables S1 and S2. Since this procedure implements simple deterministic rules and data assembly, these values do not consider uncertainty and a single figure is presented for each strata per country.

*Estimating populations at risk of low, medium, and high stable transmission*

The procedure for estimating populations at risk under differing levels of stable transmission is described in full in the main manuscript. Briefly, a previously defined Bayesian space-time geostatistical model [3] was implemented in a joint simulation framework to generate realisations of *P. falciparum* infection prevalence for the 2 up to 10 y age range (*Pf*PR_2-10_) across a 5 x 5 km grid using a data assembly of 7,953 community parasite rate surveys conducted worldwide between 1985 and 2007. Each realisation was combined with the GRUMP 2007 population surface to provide a realisation of population totals per country living under three different levels of transmission risk: *Pf*PR_2-10_ less than 5%, between 5% and 40% and greater than 40%. The relevance of these transmission thresholds for malaria management, control, and elimination policy is explained elsewhere [5]. Generating many realisations allowed posterior predictive distributions to be constructed for the total population per country living under each risk class. Figure S1 provides examples of three such posterior predictive distributions for Liberia. These distributions provide a rich source of information about the uncertainty in the modelled output. Communicating effectively that information to decision makers necessitates the extraction of summary measures from each distribution. Of primary interest are (i) a 'point estimate' that provides a measure of the central tendency of each posterior distribution and can be interpreted as the 'best estimate' for the modelled outcome and (ii) an accompanying measure of uncertainty associated with the estimate. In this instance, the arithmetic mean of each posterior distribution was chosen as the point estimate and the inter-quartile range (IQR) was chosen as a metric of uncertainty, primarily because it places less emphasis on the extreme tails of the distribution providing a more robust comparative measure of model precision. As can be seen in Figure S1, where posterior distributions are heavily skewed there is no *a priori* guarantee that the IQR will include the posterior mean.

**S1.2 Results**

Tables S1 and S2 display the population at risk estimates for each of the 80 *Pf*MECs considered. Table S1 displays absolute estimates (the number of people living in each risk strata in each country) whilst Table S2 displays relative estimates (the proportion of each country's population living in each risk strata). A single figure (with no uncertainty measurement) is provided per country for the broad stratification of population into risk-free, unstable, or stable transmission areas, whilst the presentations of populations at risk in areas into low, medium, and high stable risk include as an uncertainty metric the inter-quartile range of each posterior predictive distribution, as explained above.


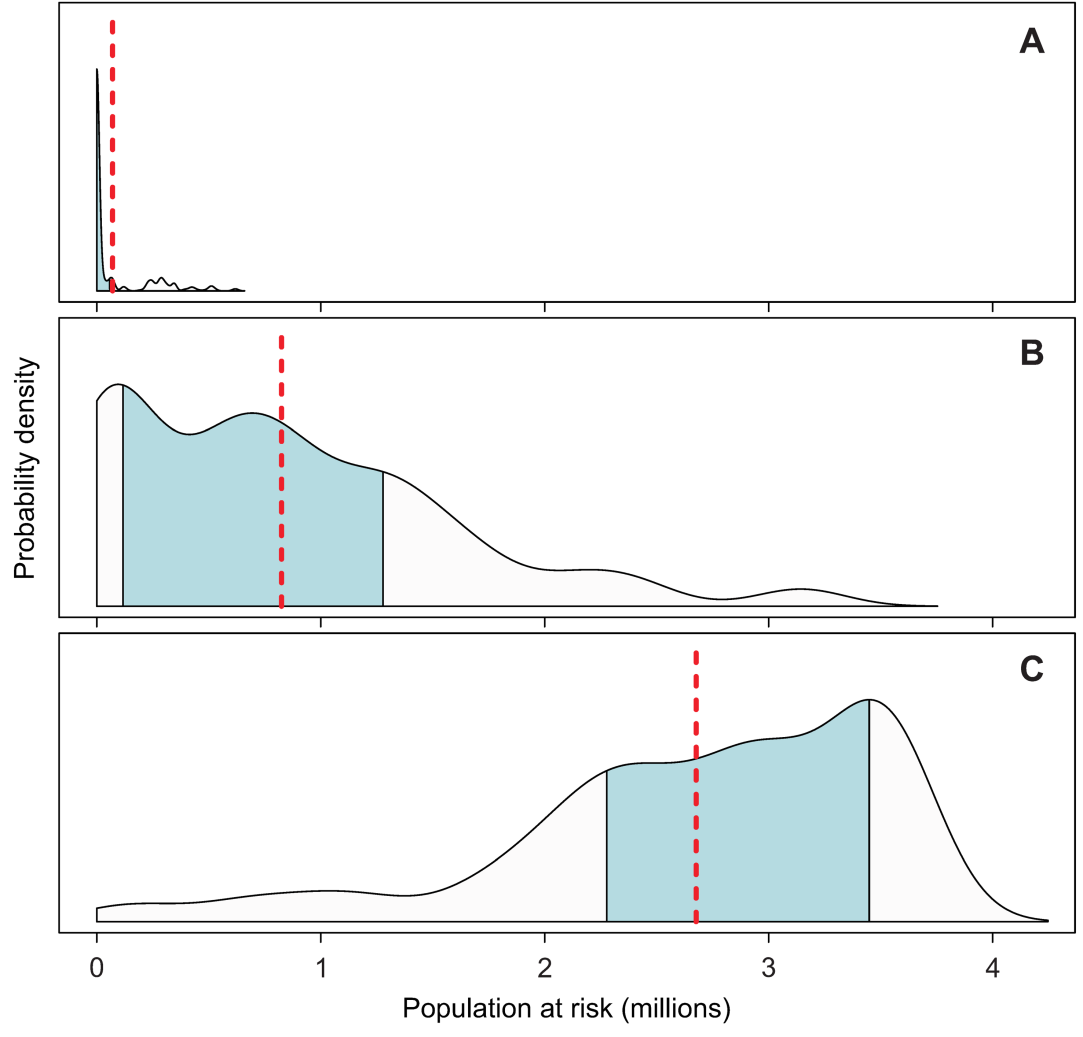


**Figure S1. Posterior predictive distributions for populations at risk under three different levels of stable transmission, the example of Liberia, West Africa.** These plots display the set of modelled realisations for the population within Liberia living under conditions of *Pf*PR_2-10_ (A) ≤5%, (B) >5% ≤40% and (C) >40% displayed as probability density plots. Shaded blue regions indicate the inter-quartile range and the dotted red line indicates the mean. The plot for population at risk of *Pf*PR_2-10_ ≤5% provides an example of the posterior mean lying outside the inter-quartile range, which can occur when distributions are strongly skewed.

| **Table S1 (part 1): Estimated populations at risk under different levels of *Plasmodium falciparum* malaria transmission intensity for 80 endemic countries^1^** | | | | | | | |
| --- | --- | --- | --- | --- | --- | --- | --- |
| **Country** | **Total population^2^** | **Risk free^3^** | **Unstable transmission**  **IR <0.1/1000 p.a^3^** | **Stable**  **transmission**  **IR >0.1/1000 p.a^3^** | **Low stable^4^**  ***Pf*PR_2-10_ ≤5% (IQR^5^)** | **Medium stable^4^**  ***Pf*PR_2-10_ >5 ≤40% (IQR^5^)** | **High stable^4^**  ***Pf*PR_2-10_ >40% (IQR^5^)** |
|  |  |  |  |  |  |  |  |
| **AFRO countries^6^** |  |  |  |  |  |  |  |
| Angola | 16,062,525 | 283,767 | 307,016 | 15,471,742 | 2,451,513 ( 614,312 - 4,065,062 ) | 5,842,131 ( 4,550,195 - 7,367,491 ) | 7,178,097 ( 4,515,458 - 10,025,490 ) |
| Benin | 7,829,101 | 0 | 0 | 7,829,101 | 416,303 ( 0 - 322,689 ) | 2,595,801 ( 1,859,461 - 3,319,692 ) | 4,816,997 ( 3,669,222 - 5,941,265 ) |
| Botswana | 1,676,977 | 784,730 | 17 | 892,230 | 706,187 ( 634,037 - 827,442 ) | 163,871 ( 60,031 - 253,856 ) | 22,172 ( 465 - 26,227 ) |
| Burkina Faso | 14,254,926 | 0 | 64 | 14,254,862 | 135,190 ( 0 - 156,159 ) | 2,519,208 ( 759,168 - 3,851,385 ) | 11,600,464 ( 10,369,286 - 13,468,808 ) |
| Burundi | 8,097,128 | 2,372,881 | 0 | 5,724,247 | 570,569 ( 4,640 - 860,478 ) | 3,315,238 ( 2,644,294 - 4,091,133 ) | 1,838,439 ( 667,528 - 2,610,308 ) |
| Cameroon | 17,378,327 | 307,628 | 0 | 17,070,699 | 1,094,071 ( 279,917 - 1,705,530 ) | 6,637,838 ( 4,856,726 - 8,659,823 ) | 9,338,791 ( 7,538,309 - 11,536,471 ) |
| Cape Verde | 502,078 | 229,257 | 272,821 | 0 | 0 ( 0 - 0 ) | 0 ( 0 - 0 ) | 0 ( 0 - 0 ) |
| Central African Republic | 4,171,345 | 0 | 0 | 4,171,345 | 362,083 ( 80,741 - 529,259 ) | 1,544,541 ( 860,320 - 2,152,652 ) | 2,264,721 ( 1,640,404 - 3,227,902 ) |
| Chad | 9,976,200 | 46 | 162,712 | 9,813,442 | 3,053,160 ( 1,813,363 - 4,066,062 ) | 4,572,872 ( 3,584,581 - 5,572,608 ) | 2,187,411 ( 765,660 - 3,443,659 ) |
| Comoros | 663,169 | 18,449 | 0 | 644,720 | 378,242 ( 208,968 - 640,156 ) | 222,310 ( 4,564 - 359,704 ) | 44,167 ( 0 - 15,430 ) |
| Congo | 3,577,086 | 0 | 48 | 3,577,038 | 144,170 ( 1,268 - 161,652 ) | 942,828 ( 247,629 - 1,509,625 ) | 2,490,040 ( 1,907,427 - 3,329,409 ) |
| Cote d'Ivoire | 18,116,585 | 36 | 0 | 18,116,549 | 97,371 ( 0 - 102,477 ) | 2,641,936 ( 1,130,101 - 3,781,081 ) | 15,377,242 ( 14,237,089 - 16,986,449 ) |
| Democratic Republic of the Congo | 62,937,744 | 3,887,571 | 191 | 59,049,982 | 5,881,787 ( 2,932,759 - 7,844,002 ) | 21,011,476 ( 17,716,976 - 24,401,257 ) | 32,156,719 ( 27,191,290 - 38,047,923 ) |
| Equatorial Guinea | 537,245 | 3,824 | 0 | 533,421 | 80 ( 0 - 0 ) | 121,902 ( 110,333 - 135,466 ) | 411,439 ( 397,954 - 423,088 ) |
| Eritrea | 4,854,578 | 548,579 | 961,100 | 3,344,899 | 2,810,861 ( 2,549,358 - 3,252,365 ) | 516,853 ( 92,534 - 793,710 ) | 17,185 ( 0 - 2,211 ) |
| Ethiopia | 75,239,839 | 27,538,597 | 1,498,369 | 46,202,873 | 31,545,737 ( 27,089,846 - 37,175,170 ) | 12,923,871 ( 8,871,079 - 17,235,537 ) | 1,733,266 ( 330,524 - 1,746,723 ) |
| Gabon | 1,385,410 | 0 | 0 | 1,385,410 | 89,989 ( 562 - 116,284 ) | 656,546 ( 461,731 - 880,666 ) | 638,876 ( 413,614 - 798,799 ) |
| Gambia | 1,599,387 | 0 | 0 | 1,599,387 | 367,030 ( 10,008 - 689,009 ) | 908,646 ( 685,620 - 1,195,097 ) | 323,710 ( 72,521 - 555,005 ) |
| Ghana | 22,470,098 | 0 | 0 | 22,470,098 | 693,014 ( 0 - 890,957 ) | 7,496,931 ( 4,676,254 - 10,429,295 ) | 14,280,153 ( 10,759,032 - 17,793,844 ) |
| Guinea | 9,338,940 | 0 | 0 | 9,338,940 | 493,093 ( 8,325 - 692,412 ) | 3,566,213 ( 2,870,155 - 4,267,815 ) | 5,279,633 ( 4,292,590 - 6,388,775 ) |
| Guinea-Bissau | 1,484,326 | 436 | 0 | 1,483,890 | 261,411 ( 3,093 - 440,815 ) | 794,556 ( 488,565 - 1,134,131 ) | 427,924 ( 91,365 - 641,426 ) |
| Kenya | 36,828,130 | 10,718,956 | 174,095 | 25,935,079 | 13,709,648 ( 13,004,842 - 14,456,225 ) | 10,422,503 ( 9,692,403 - 11,074,419 ) | 1,802,927 ( 1,318,317 - 2,059,675 ) |
| Liberia | 3,572,185 | 0 | 0 | 3,572,185 | 70,839 ( 0 - 58,110 ) | 825,065 ( 118,955 - 1,283,983 ) | 2,676,282 ( 2,283,944 - 3,453,230 ) |
| Madagascar | 19,402,242 | 1,875,347 | 0 | 17,526,895 | 3,092,491 ( 1,316,548 - 4,520,347 ) | 7,468,848 ( 6,400,360 - 8,820,776 ) | 6,965,556 ( 5,014,055 - 8,200,045 ) |
| Malawi | 13,507,165 | 1,489 | 0 | 13,505,676 | 2,192,799 ( 668,133 - 2,763,684 ) | 6,633,639 ( 5,610,842 - 7,659,091 ) | 4,679,239 ( 2,274,643 - 6,537,084 ) |
| Mali | 14,028,967 | 9 | 467,763 | 13,561,195 | 1,015,085 ( 343,712 - 1,305,949 ) | 4,213,805 ( 3,093,714 - 5,012,395 ) | 8,332,305 ( 6,610,181 - 9,727,041 ) |
| Mauritania | 1,416,147 | 71,504 | 405,639 | 939,004 | 475,803 ( 358,994 - 623,427 ) | 356,141 ( 267,637 - 423,031 ) | 107,060 ( 36,376 - 156,635 ) |
| Mayotte | 305,768 | 0 | 305,768 | 0 | 0 ( 0 - 0 ) | 0 ( 0 - 0 ) | 0 ( 0 - 0 ) |
| Mozambique | 21,498,190 | 15,758 | 0 | 21,482,432 | 3,753,610 ( 1,627,691 - 4,943,811 ) | 9,050,589 ( 7,594,643 - 10,898,726 ) | 8,678,233 ( 6,317,329 - 11,672,053 ) |
| Namibia | 1,935,760 | 298,698 | 385,841 | 1,251,221 | 719,843 ( 492,398 - 969,114 ) | 421,108 ( 255,631 - 603,936 ) | 110,270 ( 8,762 - 149,950 ) |
| Niger | 13,851,054 | 0 | 618,409 | 13,232,645 | 1,656,221 ( 359,379 - 2,438,202 ) | 5,789,797 ( 4,439,571 - 7,335,000 ) | 5,786,626 ( 3,368,141 - 8,027,651 ) |
| Nigeria | 134,996,499 | 138 | 0 | 134,996,361 | 6,890,382 ( 1,323,424 - 9,336,556 ) | 43,357,229 ( 34,028,755 - 50,577,111 ) | 84,748,749 ( 73,607,964 - 97,211,122 ) |
| Rwanda | 9,082,054 | 3,940,379 | 0 | 5,141,675 | 619,431 ( 620 - 986,324 ) | 3,045,029 ( 2,005,410 - 4,157,534 ) | 1,477,215 ( 242,658 - 2,588,512 ) |
| Sao Tome and Principe | 154,905 | 5,612 | 0 | 149,293 | 16,272 ( 0 - 9,701 ) | 86,213 ( 50,236 - 133,432 ) | 46,808 ( 0 - 79,950 ) |
| Senegal | 11,265,479 | 0 | 0 | 11,265,479 | 3,122,128 ( 1,143,442 - 4,903,535 ) | 6,164,159 ( 4,818,669 - 7,537,551 ) | 1,979,192 ( 929,295 - 2,916,298 ) |
| Sierra Leone | 5,670,712 | 0 | 0 | 5,670,712 | 220,239 ( 0 - 87,026 ) | 1,801,727 ( 516,210 - 2,823,358 ) | 3,648,746 ( 2,354,036 - 5,154,502 ) |
| Swaziland | 994,904 | 767,050 | 224 | 227,630 | 113,367 ( 9,327 - 218,532 ) | 88,414 ( 9,097 - 154,977 ) | 25,848 ( 0 - 25,323 ) |
| Togo | 5,502,409 | 0 | 0 | 5,502,409 | 159,445 ( 0 - 58,111 ) | 1,413,275 ( 427,477 - 2,076,727 ) | 3,929,689 ( 2,913,549 - 5,074,932 ) |
| Uganda | 29,100,972 | 1,887,425 | 0 | 27,213,547 | 2,151,274 ( 721,965 - 2,479,021 ) | 15,575,959 ( 14,223,879 - 17,095,281 ) | 9,486,314 ( 5,980,880 - 11,996,716 ) |
| United Republic of Tanzania | 41,935,741 | 1,592,490 | 0 | 40,343,251 | 11,854,096 ( 9,386,832 - 14,162,026 ) | 18,105,316 ( 15,999,406 - 20,125,027 ) | 10,383,839 ( 7,714,352 - 12,616,465 ) |
| Zambia | 11,902,550 | 643 | 0 | 11,901,907 | 3,599,552 ( 2,270,415 - 4,908,989 ) | 5,891,223 ( 4,979,531 - 6,743,471 ) | 2,411,132 ( 1,293,824 - 3,120,595 ) |
| Zimbabwe | 13,337,184 | 5,931,473 | 0 | 7,405,711 | 5,824,264 ( 5,145,904 - 6,720,839 ) | 1,328,415 ( 618,969 - 1,914,939 ) | 253,033 ( 6,061 - 119,590 ) |
|  |  |  |  |  |  |  |  |
|  |  |  |  |  |  |  | **Cont overleaf...** |

| **Table S1 (part 2): Estimated populations at risk under different levels of *Plasmodium falciparum* malaria transmission intensity for 80 endemic countries^1^** | | | | | | | |
| --- | --- | --- | --- | --- | --- | --- | --- |
| **Country** | **Total population^2^** | **Risk free^3^** | **Unstable transmission**  **IR <0.1/1000 p.a^3^** | **Stable**  **transmission**  **IR >0.1/1000 p.a^3^** | **Low stable^4^**  ***Pf*PR_2-10_ ≤5% (IQR^5^)** | **Medium stable^4^**  ***Pf*PR_2-10_ >5 ≤40% (IQR^5^)** | **High stable^4^**  ***Pf*PR_2-10_ >40% (IQR^5^)** |
|  |  |  |  |  |  |  |  |
| **AMRO countries^6^** |  |  |  |  |  |  |  |
| Bolivia | 9,525,542 | 6,691,342 | 2,614,390 | 219,810 | 216,163 ( 219,720 - 219,810 ) | 3,647 ( 0 - 90 ) | 0 ( 0 - 0 ) |
| Brazil | 187,408,605 | 155,568,917 | 18,751,830 | 13,087,858 | 11,590,102 ( 11,021,012 - 12,424,357 ) | 1,463,210 ( 663,132 - 1,678,061 ) | 34,545 ( 0 - 2,246 ) |
| Colombia | 46,565,277 | 31,330,913 | 9,918,929 | 5,315,435 | 4,457,080 ( 4,077,393 - 5,278,384 ) | 835,578 ( 37,051 - 1,238,042 ) | 22,778 ( 0 - 6 ) |
| Dominican Republic | 9,336,959 | 5,023,902 | 2,885,597 | 1,427,460 | 1,296,192 ( 1,273,405 - 1,426,412 ) | 128,586 ( 1,048 - 154,055 ) | 2,682 ( 0 - 0 ) |
| Ecuador | 13,767,590 | 7,899,476 | 1,659,491 | 4,208,623 | 4,044,253 ( 4,066,548 - 4,208,623 ) | 164,370 ( 0 - 142,075 ) | 0 ( 0 - 0 ) |
| French Guiana | 200,125 | 58,200 | 0 | 141,925 | 127,133 ( 124,081 - 141,922 ) | 14,761 ( 3 - 17,844 ) | 32 ( 0 - 0 ) |
| Guatemala | 13,492,957 | 7,129,638 | 5,338,224 | 1,025,095 | 989,330 ( 1,024,772 - 1,025,095 ) | 22,792 ( 0 - 322 ) | 12,973 ( 0 - 0 ) |
| Guyana | 762,160 | 93,363 | 528,427 | 140,370 | 139,351 ( 140,345 - 140,370 ) | 1,019 ( 0 - 25 ) | 0 ( 0 - 0 ) |
| Haiti | 9,111,843 | 213,827 | 0 | 8,898,016 | 7,427,746 ( 6,190,107 - 8,826,472 ) | 1,449,709 ( 71,544 - 2,707,908 ) | 20,560 ( 0 - 0 ) |
| Honduras | 7,352,209 | 3,803,089 | 2,631,892 | 917,228 | 882,845 ( 894,575 - 917,228 ) | 34,383 ( 0 - 22,653 ) | 0 ( 0 - 0 ) |
| Nicaragua | 5,565,397 | 1,861,479 | 2,126,458 | 1,577,460 | 1,523,595 ( 1,574,966 - 1,577,460 ) | 53,865 ( 0 - 2,494 ) | 0 ( 0 - 0 ) |
| Peru | 27,783,689 | 22,190,478 | 1,729,653 | 3,863,558 | 3,689,075 ( 3,611,245 - 3,860,649 ) | 174,458 ( 2,910 - 252,314 ) | 25 ( 0 - 0 ) |
| Suriname | 438,416 | 379,303 | 52,200 | 6,913 | 6,532 ( 6,560 - 6,913 ) | 381 ( 0 - 354 ) | 0 ( 0 - 0 ) |
| Venezuela | 27,379,619 | 20,845,532 | 6,313,150 | 220,937 | 194,329 ( 192,726 - 220,912 ) | 26,607 ( 25 - 28,211 ) | 1 ( 0 - 0 ) |
|  |  |  |  |  |  |  |  |
| **EMRO countries^6^** |  |  |  |  |  |  |  |
| Afghanistan | 28,428,464 | 11,304,634 | 12,545,338 | 4,578,492 | 3,448,678 ( 2,894,406 - 4,171,002 ) | 997,354 ( 407,490 - 1,548,407 ) | 132,460 ( 0 - 50,104 ) |
| Djibouti | 721,101 | 250,723 | 448,120 | 22,258 | 14,457 ( 7,392 - 22,258 ) | 7,109 ( 0 - 14,112 ) | 692 ( 0 - 93 ) |
| Pakistan | 160,455,248 | 60,342,227 | 68,890,127 | 31,222,894 | 26,469,571 ( 24,357,947 - 30,041,826 ) | 4,018,185 ( 1,175,623 - 6,286,124 ) | 735,138 ( 0 - 455,804 ) |
| Somalia | 10,812,680 | 3,554 | 550,185 | 10,258,941 | 5,221,397 ( 4,790,611 - 5,578,553 ) | 4,283,054 ( 3,991,319 - 4,708,082 ) | 754,491 ( 482,439 - 903,661 ) |
| Sudan | 35,953,706 | 4,111 | 6,870,242 | 29,079,353 | 17,314,132 ( 14,626,344 - 20,694,012 ) | 9,818,840 ( 7,711,986 - 11,829,200 ) | 1,946,380 ( 827,981 - 2,909,070 ) |
| Yemen | 22,592,191 | 1,326,416 | 5,486,926 | 15,778,849 | 7,135,920 ( 4,409,915 - 9,227,603 ) | 7,636,451 ( 6,320,313 - 9,311,722 ) | 1,006,478 ( 193,495 - 1,406,794 ) |
|  |  |  |  |  |  |  |  |
| **SEARO countries^6^** |  |  |  |  |  |  |  |
| Bangladesh | 155,854,154 | 91,456,889 | 48,985,652 | 15,411,613 | 4,539,660 ( 947,925 - 8,050,948 ) | 6,268,730 ( 2,437,205 - 9,525,201 ) | 4,603,223 ( 246,077 - 7,971,577 ) |
| Bhutan | 2,404,565 | 1,132,905 | 464,789 | 806,871 | 602,766 ( 542,380 - 800,566 ) | 128,886 ( 6,305 - 129,608 ) | 75,219 ( 0 - 0 ) |
| India | 1,107,527,880 | 98,276,063 | 602,805,910 | 406,445,907 | 264,413,567 ( 220,584,751 - 315,387,090 ) | 106,871,952 ( 78,537,252 - 130,414,710 ) | 35,160,388 ( 12,572,269 - 53,254,289 ) |
| Indonesia | 231,739,917 | 74,865,098 | 86,126,495 | 70,748,324 | 52,661,527 ( 49,206,636 - 55,444,558 ) | 14,808,149 ( 11,359,604 - 17,241,114 ) | 3,278,647 ( 2,199,007 - 3,965,066 ) |
| Myanmar | 50,797,944 | 177,639 | 136 | 50,620,169 | 16,370,527 ( 11,268,208 - 21,919,252 ) | 22,891,887 ( 19,530,440 - 26,081,653 ) | 11,357,754 ( 5,770,453 - 16,022,138 ) |
| Nepal | 26,608,915 | 16,968,407 | 6,224,395 | 3,416,113 | 3,403,671 ( 3,416,113 - 3,416,113 ) | 12,442 ( 0 - 0 ) | 0 ( 0 - 0 ) |
| Sri Lanka | 19,515,907 | 9,843,380 | 7,768,834 | 1,903,693 | 1,395,981 ( 1,165,276 - 1,893,972 ) | 440,080 ( 9,721 - 738,417 ) | 67,633 ( 0 - 13,369 ) |
| Thailand | 65,889,878 | 17,587,279 | 31,394,831 | 16,907,768 | 11,322,175 ( 9,615,629 - 13,444,667 ) | 4,359,321 ( 2,997,783 - 6,024,080 ) | 1,226,272 ( 232,397 - 1,713,317 ) |
| Timor-Leste | 789,820 | 29,430 | 0 | 760,390 | 404,812 ( 198,491 - 609,273 ) | 260,789 ( 101,675 - 443,727 ) | 94,788 ( 0 - 127,727 ) |
|  |  |  |  |  |  |  |  |
| **WPRO countries^6^** |  |  |  |  |  |  |  |
| Cambodia | 14,811,574 | 1,337,630 | 2,602,863 | 10,871,081 | 6,515,119 ( 4,381,903 - 9,073,668 ) | 3,757,587 ( 1,741,089 - 5,030,157 ) | 598,375 ( 31,484 - 397,106 ) |
| China | 1,327,387,933 | 1,289,024,597 | 20,365,592 | 17,997,744 | 15,851,709 ( 14,863,732 - 17,332,576 ) | 1,951,450 ( 662,370 - 3,079,791 ) | 194,585 ( 3,785 - 116,876 ) |
| Lao People's Democratic Republic | 5,932,112 | 611,181 | 37,192 | 5,283,739 | 2,865,337 ( 2,450,729 - 3,358,482 ) | 1,812,020 ( 1,417,560 - 2,137,014 ) | 606,383 ( 222,179 - 855,493 ) |
| Malaysia | 25,291,534 | 2,390,213 | 16,476,967 | 6,424,354 | 4,005,839 ( 3,107,852 - 4,712,460 ) | 1,795,254 ( 1,242,172 - 2,248,777 ) | 623,261 ( 129,457 - 1,008,299 ) |
| Papua New Guinea | 5,645,661 | 1,413,630 | 0 | 4,232,031 | 2,018,775 ( 1,604,751 - 2,419,352 ) | 1,594,005 ( 1,417,977 - 1,832,421 ) | 619,251 ( 392,276 - 844,122 ) |
| Philippines | 86,775,400 | 38,435,250 | 20,896,063 | 27,444,087 | 19,644,764 ( 15,158,220 - 23,687,086 ) | 5,791,026 ( 3,282,918 - 7,842,408 ) | 2,008,298 ( 157,642 - 2,592,009 ) |
| Solomon islands | 532,875 | 24,174 | 0 | 508,701 | 194,498 ( 51,730 - 337,224 ) | 168,690 ( 91,967 - 254,493 ) | 145,512 ( 28,604 - 272,337 ) |
| Vanuatu | 234,304 | 116 | 0 | 234,188 | 178,683 ( 145,397 - 219,710 ) | 51,061 ( 14,478 - 85,653 ) | 4,444 ( 0 - 3,399 ) |
| Viet Nam | 86,221,820 | 10,293,049 | 54,815,022 | 21,113,749 | 14,972,876 ( 14,259,985 - 17,165,290 ) | 5,567,426 ( 3,862,358 - 6,454,617 ) | 573,447 ( 104,521 - 770,426 ) |
|  |  |  |  |  |  |  |  |
|  |  |  |  |  |  |  |  |
| 1. 87 countries have been defined previously as endemic for *Plasmodium falciparum* malaria [4,4], of which seven (South Africa, Saudi Arabia, Belize, Panama, Iran, Kyrgyzstan, Tajikistan) were excluded from these analyses as representing very low transmission intensity and gold-standard health system reporting not requiring modelled population at risk estimates. | | | | | | | |
| 2. Population data were obtained for the year 2000 from the Global Rural Urban Mapping Project (GRUMP) alpha version and projected to 2007 by applying United Nations national, medium variant, inter-censal growth rates by country [1,2] | | | | | | | |
| 3. A combination of nationally reported case incidence data, medical intelligence, and biological rules on the constraint on transmission of temperature and aridity were used to classify regions in to three broad classes of transmission intensity: No risk (no cases likely to be reported over several years), unstable transmission (annual case incidence (or incidence rate, (IR)) likely to be less than 1 in 10,000), and stable transmission (IR likely to be greater than 1 in 10,000), as presented previously [4].  4. Estimation of populations living under low, medium, and high stable transmission risk was achieved via a Bayesian space-time geostatistical model implemented via joint simulation as presented in the current study.  5. IQR: inter-quartile range of the posterior predictive distribution for each population at risk estimate. Where posterior distributions are heavily skewed, the mean estimate does not necessarily lie within the IQR.  6. Countries grouped by World Health Organisation Regional Offices: AFRO, African; SEARO, South East Asian; WPRO, Western Pacific; EMRO, Eastern Mediterranean; AMRO, American; EURO, European. | | | | | | | |
|  |  |  |  |  |  |  |  |
| **Table S2 (part 1): Estimated percentages of population at risk under different levels of *Plasmodium falciparum* malaria transmission intensity for 80 endemic countries^1^** | | | | | | | |
| **Country** | **Total population^2^** | **Risk free^3^** | **Unstable transmission**  **IR <0.1/1000 p.a^3^** | **Stable**  **transmission**  **IR >0.1/1000 p.a^3^** | **Low stable^4^**  ***Pf*PR_2-10_ ≤5% (IQR^5^)** | **Medium stable^4^**  ***Pf*PR_2-10_ >5 ≤40% (IQR^5^)** | **High stable^4^**  ***Pf*PR_2-10_ >40% (IQR^5^)** |
|  |  |  |  |  |  |  |  |
| **AFRO countries^6^** |  |  |  |  |  |  |  |
| Angola | 100.00 | 1.77 | 1.91 | 96.32 | 15.26 ( 3.82 - 25.31 ) | 36.37 ( 28.33 - 45.87 ) | 44.69 ( 28.11 - 62.42 ) |
| Benin | 100.00 | 0.00 | 0.00 | 100.00 | 5.32 ( 0.00 - 4.12 ) | 33.16 ( 23.75 - 42.40 ) | 61.53 ( 46.87 - 75.89 ) |
| Botswana | 100.00 | 46.79 | 0.00 | 53.20 | 42.11 ( 37.81 - 49.34 ) | 9.77 ( 3.58 - 15.14 ) | 1.32 ( 0.03 - 1.56 ) |
| Burkina Faso | 100.00 | 0.00 | 0.00 | 100.00 | 0.95 ( 0.00 - 1.10 ) | 17.67 ( 5.33 - 27.02 ) | 81.38 ( 72.74 - 94.49 ) |
| Burundi | 100.00 | 29.31 | 0.00 | 70.69 | 7.05 ( 0.06 - 10.63 ) | 40.94 ( 32.66 - 50.53 ) | 22.70 ( 8.24 - 32.24 ) |
| Cameroon | 100.00 | 1.77 | 0.00 | 98.23 | 6.30 ( 1.61 - 9.81 ) | 38.20 ( 27.95 - 49.83 ) | 53.74 ( 43.38 - 66.38 ) |
| Cape Verde | 100.00 | 45.66 | 54.34 | 0.00 | 0.00 ( 0.00 - 0.00 ) | 0.00 ( 0.00 - 0.00 ) | 0.00 ( 0.00 - 0.00 ) |
| Central African Republic | 100.00 | 0.00 | 0.00 | 100.00 | 8.68 ( 1.94 - 12.69 ) | 37.03 ( 20.62 - 51.61 ) | 54.29 ( 39.33 - 77.38 ) |
| Chad | 100.00 | 0.00 | 1.63 | 98.37 | 30.60 ( 18.18 - 40.76 ) | 45.84 ( 35.93 - 55.86 ) | 21.93 ( 7.67 - 34.52 ) |
| Comoros | 100.00 | 2.78 | 0.00 | 97.22 | 57.04 ( 31.51 - 96.53 ) | 33.52 ( 0.69 - 54.24 ) | 6.66 ( 0.00 - 2.33 ) |
| Congo | 100.00 | 0.00 | 0.00 | 100.00 | 4.03 ( 0.04 - 4.52 ) | 26.36 ( 6.92 - 42.20 ) | 69.61 ( 53.32 - 93.08 ) |
| Cote d'Ivoire | 100.00 | 0.00 | 0.00 | 100.00 | 0.54 ( 0.00 - 0.57 ) | 14.58 ( 6.24 - 20.87 ) | 84.88 ( 78.59 - 93.76 ) |
| Democratic Republic of the Congo | 100.00 | 6.18 | 0.00 | 93.82 | 9.35 ( 4.66 - 12.46 ) | 33.38 ( 28.15 - 38.77 ) | 51.09 ( 43.20 - 60.45 ) |
| Equatorial Guinea | 100.00 | 0.71 | 0.00 | 99.29 | 0.01 ( 0.00 - 0.00 ) | 22.69 ( 20.54 - 25.22 ) | 76.58 ( 74.07 - 78.75 ) |
| Eritrea | 100.00 | 11.30 | 19.80 | 68.90 | 57.90 ( 52.51 - 67.00 ) | 10.65 ( 1.91 - 16.35 ) | 0.35 ( 0.00 - 0.05 ) |
| Ethiopia | 100.00 | 36.60 | 1.99 | 61.41 | 41.93 ( 36.00 - 49.41 ) | 17.18 ( 11.79 - 22.91 ) | 2.30 ( 0.44 - 2.32 ) |
| Gabon | 100.00 | 0.00 | 0.00 | 100.00 | 6.50 ( 0.04 - 8.39 ) | 47.39 ( 33.33 - 63.57 ) | 46.11 ( 29.85 - 57.66 ) |
| Gambia | 100.00 | 0.00 | 0.00 | 100.00 | 22.95 ( 0.63 - 43.08 ) | 56.81 ( 42.87 - 74.72 ) | 20.24 ( 4.53 - 34.70 ) |
| Ghana | 100.00 | 0.00 | 0.00 | 100.00 | 3.08 ( 0.00 - 3.97 ) | 33.36 ( 20.81 - 46.41 ) | 63.55 ( 47.88 - 79.19 ) |
| Guinea | 100.00 | 0.00 | 0.00 | 100.00 | 5.28 ( 0.09 - 7.41 ) | 38.19 ( 30.73 - 45.70 ) | 56.53 ( 45.96 - 68.41 ) |
| Guinea-Bissau | 100.00 | 0.03 | 0.00 | 99.97 | 17.61 ( 0.21 - 29.70 ) | 53.53 ( 32.91 - 76.41 ) | 28.83 ( 6.16 - 43.21 ) |
| Kenya | 100.00 | 29.11 | 0.47 | 70.42 | 37.23 ( 35.31 - 39.25 ) | 28.30 ( 26.32 - 30.07 ) | 4.90 ( 3.58 - 5.59 ) |
| Liberia | 100.00 | 0.00 | 0.00 | 100.00 | 1.98 ( 0.00 - 1.63 ) | 23.10 ( 3.33 - 35.94 ) | 74.92 ( 63.94 - 96.67 ) |
| Madagascar | 100.00 | 9.67 | 0.00 | 90.33 | 15.94 ( 6.79 - 23.30 ) | 38.49 ( 32.99 - 45.46 ) | 35.90 ( 25.84 - 42.26 ) |
| Malawi | 100.00 | 0.01 | 0.00 | 99.99 | 16.23 ( 4.95 - 20.46 ) | 49.11 ( 41.54 - 56.70 ) | 34.64 ( 16.84 - 48.40 ) |
| Mali | 100.00 | 0.00 | 3.33 | 96.67 | 7.24 ( 2.45 - 9.31 ) | 30.04 ( 22.05 - 35.73 ) | 59.39 ( 47.12 - 69.34 ) |
| Mauritania | 100.00 | 5.05 | 28.64 | 66.31 | 33.60 ( 25.35 - 44.02 ) | 25.15 ( 18.90 - 29.87 ) | 7.56 ( 2.57 - 11.06 ) |
| Mayotte | 100.00 | 0.00 | 100.00 | 0.00 | 0.00 ( 0.00 - 0.00 ) | 0.00 ( 0.00 - 0.00 ) | 0.00 ( 0.00 - 0.00 ) |
| Mozambique | 100.00 | 0.07 | 0.00 | 99.93 | 17.46 ( 7.57 - 23.00 ) | 42.10 ( 35.33 - 50.70 ) | 40.37 ( 29.39 - 54.29 ) |
| Namibia | 100.00 | 15.43 | 19.93 | 64.64 | 37.19 ( 25.44 - 50.06 ) | 21.75 ( 13.21 - 31.20 ) | 5.70 ( 0.45 - 7.75 ) |
| Niger | 100.00 | 0.00 | 4.46 | 95.54 | 11.96 ( 2.59 - 17.60 ) | 41.80 ( 32.05 - 52.96 ) | 41.78 ( 24.32 - 57.96 ) |
| Nigeria | 100.00 | 0.00 | 0.00 | 100.00 | 5.10 ( 0.98 - 6.92 ) | 32.12 ( 25.21 - 37.47 ) | 62.78 ( 54.53 - 72.01 ) |
| Rwanda | 100.00 | 43.39 | 0.00 | 56.61 | 6.82 ( 0.01 - 10.86 ) | 33.53 ( 22.08 - 45.78 ) | 16.27 ( 2.67 - 28.50 ) |
| Sao Tome and Principe | 100.00 | 3.62 | 0.00 | 96.38 | 10.50 ( 0.00 - 6.26 ) | 55.66 ( 32.43 - 86.14 ) | 30.22 ( 0.00 - 51.61 ) |
| Senegal | 100.00 | 0.00 | 0.00 | 100.00 | 27.71 ( 10.15 - 43.53 ) | 54.72 ( 42.77 - 66.91 ) | 17.57 ( 8.25 - 25.89 ) |
| Sierra Leone | 100.00 | 0.00 | 0.00 | 100.00 | 3.88 ( 0.00 - 1.53 ) | 31.77 ( 9.10 - 49.79 ) | 64.34 ( 41.51 - 90.90 ) |
| Swaziland | 100.00 | 77.10 | 0.02 | 22.88 | 11.39 ( 0.94 - 21.97 ) | 8.89 ( 0.91 - 15.58 ) | 2.60 ( 0.00 - 2.55 ) |
| Togo | 100.00 | 0.00 | 0.00 | 100.00 | 2.90 ( 0.00 - 1.06 ) | 25.68 ( 7.77 - 37.74 ) | 71.42 ( 52.95 - 92.23 ) |
| Uganda | 100.00 | 6.49 | 0.00 | 93.51 | 7.39 ( 2.48 - 8.52 ) | 53.52 ( 48.88 - 58.74 ) | 32.60 ( 20.55 - 41.22 ) |
| United Republic of Tanzania | 100.00 | 3.80 | 0.00 | 96.20 | 28.27 ( 22.38 - 33.77 ) | 43.17 ( 38.15 - 47.99 ) | 24.76 ( 18.40 - 30.09 ) |
| Zambia | 100.00 | 0.01 | 0.00 | 99.99 | 30.24 ( 19.08 - 41.24 ) | 49.50 ( 41.84 - 56.66 ) | 20.26 ( 10.87 - 26.22 ) |
| Zimbabwe | 100.00 | 44.47 | 0.00 | 55.53 | 43.67 ( 38.58 - 50.39 ) | 9.96 ( 4.64 - 14.36 ) | 1.90 ( 0.05 - 0.90 ) |
|  |  |  |  |  |  |  |  |
|  |  |  |  |  |  |  | **Cont overleaf...** |

| **Table S2 (part 2): Estimated percentages of population at risk under different levels of *Plasmodium falciparum* malaria transmission intensity for 80 endemic countries^1^** | | | | | | | |
| --- | --- | --- | --- | --- | --- | --- | --- |
| **Country** | **Total population^2^** | **Risk free^3^** | **Unstable transmission**  **IR <0.1/1000 p.a^3^** | **Stable**  **transmission**  **IR >0.1/1000 p.a^3^** | **Low stable^4^**  ***Pf*PR_2-10_ ≤5% (IQR^5^)** | **Medium stable^4^**  ***Pf*PR_2-10_ >5 ≤40% (IQR^5^)** | **High stable^4^**  ***Pf*PR_2-10_ >40% (IQR^5^)** |
|  |  |  |  |  |  |  |  |
| **AMRO countries^6^** |  |  |  |  |  |  |  |
| Bolivia | 100.00 | 70.25 | 27.45 | 2.31 | 2.27 ( 2.31 - 2.31 ) | 0.04 ( 0.00 - 0.00 ) | 0.00 ( 0.00 - 0.00 ) |
| Brazil | 100.00 | 83.01 | 10.01 | 6.98 | 6.18 ( 5.88 - 6.63 ) | 0.78 ( 0.35 - 0.90 ) | 0.02 ( 0.00 - 0.00 ) |
| Colombia | 100.00 | 67.28 | 21.30 | 11.42 | 9.57 ( 8.76 - 11.34 ) | 1.79 ( 0.08 - 2.66 ) | 0.05 ( 0.00 - 0.00 ) |
| Dominican Republic | 100.00 | 53.81 | 30.91 | 15.29 | 13.88 ( 13.64 - 15.28 ) | 1.38 ( 0.01 - 1.65 ) | 0.03 ( 0.00 - 0.00 ) |
| Ecuador | 100.00 | 57.38 | 12.05 | 30.57 | 29.38 ( 29.54 - 30.57 ) | 1.19 ( 0.00 - 1.03 ) | 0.00 ( 0.00 - 0.00 ) |
| French Guiana | 100.00 | 29.08 | 0.00 | 70.92 | 63.53 ( 62.00 - 70.92 ) | 7.38 ( 0.00 - 8.92 ) | 0.02 ( 0.00 - 0.00 ) |
| Guatemala | 100.00 | 52.84 | 39.56 | 7.60 | 7.33 ( 7.59 - 7.60 ) | 0.17 ( 0.00 - 0.00 ) | 0.10 ( 0.00 - 0.00 ) |
| Guyana | 100.00 | 12.25 | 69.33 | 18.42 | 18.28 ( 18.41 - 18.42 ) | 0.13 ( 0.00 - 0.00 ) | 0.00 ( 0.00 - 0.00 ) |
| Haiti | 100.00 | 2.35 | 0.00 | 97.65 | 81.52 ( 67.93 - 96.87 ) | 15.91 ( 0.79 - 29.72 ) | 0.23 ( 0.00 - 0.00 ) |
| Honduras | 100.00 | 51.73 | 35.80 | 12.48 | 12.01 ( 12.17 - 12.48 ) | 0.47 ( 0.00 - 0.31 ) | 0.00 ( 0.00 - 0.00 ) |
| Nicaragua | 100.00 | 33.45 | 38.21 | 28.34 | 27.38 ( 28.30 - 28.34 ) | 0.97 ( 0.00 - 0.04 ) | 0.00 ( 0.00 - 0.00 ) |
| Peru | 100.00 | 79.87 | 6.23 | 13.91 | 13.28 ( 13.00 - 13.90 ) | 0.63 ( 0.01 - 0.91 ) | 0.00 ( 0.00 - 0.00 ) |
| Suriname | 100.00 | 86.52 | 11.91 | 1.58 | 1.49 ( 1.50 - 1.58 ) | 0.09 ( 0.00 - 0.08 ) | 0.00 ( 0.00 - 0.00 ) |
| Venezuela | 100.00 | 76.14 | 23.06 | 0.81 | 0.71 ( 0.70 - 0.81 ) | 0.10 ( 0.00 - 0.10 ) | 0.00 ( 0.00 - 0.00 ) |
|  |  |  |  |  |  |  |  |
| **EMRO countries^6^** |  |  |  |  |  |  |  |
| Afghanistan | 100.00 | 39.77 | 44.13 | 16.11 | 12.13 ( 10.18 - 14.67 ) | 3.51 ( 1.43 - 5.45 ) | 0.47 ( 0.00 - 0.18 ) |
| Djibouti | 100.00 | 34.77 | 62.14 | 3.09 | 2.00 ( 1.03 - 3.09 ) | 0.99 ( 0.00 - 1.96 ) | 0.10 ( 0.00 - 0.01 ) |
| Pakistan | 100.00 | 37.61 | 42.93 | 19.46 | 16.50 ( 15.18 - 18.72 ) | 2.50 ( 0.73 - 3.92 ) | 0.46 ( 0.00 - 0.28 ) |
| Somalia | 100.00 | 0.03 | 5.09 | 94.88 | 48.29 ( 44.31 - 51.59 ) | 39.61 ( 36.91 - 43.54 ) | 6.98 ( 4.46 - 8.36 ) |
| Sudan | 100.00 | 0.01 | 19.11 | 80.88 | 48.16 ( 40.68 - 57.56 ) | 27.31 ( 21.45 - 32.90 ) | 5.41 ( 2.30 - 8.09 ) |
| Yemen | 100.00 | 5.87 | 24.29 | 69.84 | 31.59 ( 19.52 - 40.84 ) | 33.80 ( 27.98 - 41.22 ) | 4.45 ( 0.86 - 6.23 ) |
|  |  |  |  |  |  |  |  |
| **SEARO countries^6^** |  |  |  |  |  |  |  |
| Bangladesh | 100.00 | 58.68 | 31.43 | 9.89 | 2.91 ( 0.61 - 5.17 ) | 4.02 ( 1.56 - 6.11 ) | 2.95 ( 0.16 - 5.11 ) |
| Bhutan | 100.00 | 47.11 | 19.33 | 33.56 | 25.07 ( 22.56 - 33.29 ) | 5.36 ( 0.26 - 5.39 ) | 3.13 ( 0.00 - 0.00 ) |
| India | 100.00 | 8.87 | 54.43 | 36.70 | 23.87 ( 19.92 - 28.48 ) | 9.65 ( 7.09 - 11.78 ) | 3.17 ( 1.14 - 4.81 ) |
| Indonesia | 100.00 | 32.31 | 37.17 | 30.53 | 22.72 ( 21.23 - 23.93 ) | 6.39 ( 4.90 - 7.44 ) | 1.41 ( 0.95 - 1.71 ) |
| Myanmar | 100.00 | 0.35 | 0.00 | 99.65 | 32.23 ( 22.18 - 43.15 ) | 45.06 ( 38.45 - 51.34 ) | 22.36 ( 11.36 - 31.54 ) |
| Nepal | 100.00 | 63.77 | 23.39 | 12.84 | 12.79 ( 12.84 - 12.84 ) | 0.05 ( 0.00 - 0.00 ) | 0.00 ( 0.00 - 0.00 ) |
| Sri Lanka | 100.00 | 50.44 | 39.81 | 9.75 | 7.15 ( 5.97 - 9.70 ) | 2.25 ( 0.05 - 3.78 ) | 0.35 ( 0.00 - 0.07 ) |
| Thailand | 100.00 | 26.69 | 47.65 | 25.66 | 17.18 ( 14.59 - 20.40 ) | 6.62 ( 4.55 - 9.14 ) | 1.86 ( 0.35 - 2.60 ) |
| Timor-Leste | 100.00 | 3.73 | 0.00 | 96.27 | 51.25 ( 25.13 - 77.14 ) | 33.02 ( 12.87 - 56.18 ) | 12.00 ( 0.00 - 16.17 ) |
|  |  |  |  |  |  |  |  |
| **WPRO countries^6^** |  |  |  |  |  |  |  |
| Cambodia | 100.00 | 9.03 | 17.57 | 73.40 | 43.99 ( 29.58 - 61.26 ) | 25.37 ( 11.75 - 33.96 ) | 4.04 ( 0.21 - 2.68 ) |
| China | 100.00 | 97.11 | 1.53 | 1.36 | 1.19 ( 1.12 - 1.31 ) | 0.15 ( 0.05 - 0.23 ) | 0.01 ( 0.00 - 0.01 ) |
| Lao People's Democratic Republic | 100.00 | 10.30 | 0.63 | 89.07 | 48.30 ( 41.31 - 56.62 ) | 30.55 ( 23.90 - 36.02 ) | 10.22 ( 3.75 - 14.42 ) |
| Malaysia | 100.00 | 9.45 | 65.15 | 25.40 | 15.84 ( 12.29 - 18.63 ) | 7.10 ( 4.91 - 8.89 ) | 2.46 ( 0.51 - 3.99 ) |
| Papua New Guinea | 100.00 | 25.04 | 0.00 | 74.96 | 35.76 ( 28.42 - 42.85 ) | 28.23 ( 25.12 - 32.46 ) | 10.97 ( 6.95 - 14.95 ) |
| Philippines | 100.00 | 44.29 | 24.08 | 31.63 | 22.64 ( 17.47 - 27.30 ) | 6.67 ( 3.78 - 9.04 ) | 2.31 ( 0.18 - 2.99 ) |
| Solomon islands | 100.00 | 4.54 | 0.00 | 95.46 | 36.50 ( 9.71 - 63.28 ) | 31.66 ( 17.26 - 47.76 ) | 27.31 ( 5.37 - 51.11 ) |
| Vanuatu | 100.00 | 0.05 | 0.00 | 99.95 | 76.26 ( 62.05 - 93.77 ) | 21.79 ( 6.18 - 36.56 ) | 1.90 ( 0.00 - 1.45 ) |
| Viet Nam | 100.00 | 11.94 | 63.57 | 24.49 | 17.37 ( 16.54 - 19.91 ) | 6.46 ( 4.48 - 7.49 ) | 0.67 ( 0.12 - 0.89 ) |
|  |  |  |  |  |  |  |  |
|  |  |  |  |  |  |  |  |
| 1. 87 countries have been defined previously as endemic for *Plasmodium falciparum* malaria [4], of which seven (South Africa, Saudi Arabia, Belize, Panama, Iran, Kyrgyzstan, Tajikistan) were excluded from these analyses as representing very low transmission intensity and gold-standard health system reporting not requiring modelled population at risk estimates. | | | | | | | |
| 2. Denominator population data were obtained for the year 2000 from the Global Rural Urban Mapping Project (GRUMP) alpha version and projected to 2007 by applying United Nations national, medium variant, inter-censal growth rates by country [1,2] | | | | | | | |
| 3. A combination of nationally reported case incidence data, medical intelligence, and biological rules on the constraint on transmission of temperature and aridity were used to classify regions in to three broad classes of transmission intensity: No risk (no cases likely to be reported over several years), unstable transmission (annual case incidence (or incidence rate, (IR)) likely to be less than 1 in 10,000), and stable transmission (IR likely to be greater than 1 in 10,000), as presented previously [4].  4. Estimation of percentage populations living under low, medium, and high stable transmission risk was achieved via a Bayesian space-time geostatistical model implemented via joint simulation as presented in the current study.  5. IQR: inter-quartile range of the posterior predictive distribution for each percentage population at risk estimate. Where posterior distributions are heavily skewed, the mean estimate does not necessarily lie within the IQR.  6. Countries grouped by World Health Organisation Regional Offices: AFRO, African; SEARO, South East Asian; WPRO, Western Pacific; EMRO, Eastern Mediterranean; AMRO, American; EURO, European. | | | | | | | |

**References**

1. Balk DL, Deichmann U, Yetman G, Pozzi F, Hay SI et al. (2006) Determining global population distribution: methods, applications and data. Adv Parasitol 62: 119-156.

2. U.N.P.D. (2006) World population prospects: the 2006 revision population database. http://esa.un.org/unpp/. New York: United Nations Population Division (U.N.D.P).

3. Hay SI, Guerra CA, Gething PW, Patil AP, Tatem AJ et al. (2009) A world malaria map: *Plasmodium falciparum* endemicity in 2007. PLoS Med 6: e1000048.

4. Guerra CA, Gikandi PW, Tatem AJ, Noor AM, Smith DL et al. (2008) The limits and intensity of *Plasmodium falciparum* transmission: implications for malaria control and elimination worldwide. PLoS Med 5: e38.

5. Hay SI, Smith DL, Snow RW (2008) Measuring malaria endemicity from intense to interrupted transmission. Lancet Infect Dis 8: 369-378.
